# A Triple-Port High-Pressure Volumetric Sorption Analyzer: Calibration, Measurements, and Uncertainty Analysis for Refrigerant Blend Separation Applications

**DOI:** 10.1021/acsomega.5c09911

**Published:** 2026-03-06

**Authors:** Gensheng Lin, Panpan Liu, Markus Richter, Xiaoxian Yang

**Affiliations:** † Applied Thermodynamics, 38869 Chemnitz University of Technology, 09107 Chemnitz, Germany; ‡ Institute of Thermodynamics, Leibniz University Hannover, An der Universität 1, 30823 Garbsen, Germany; § College of Pharmacy, Heze University, Heze 274015, PR China

## Abstract

A triple-port automatic high-pressure volumetric sorption analyzer was set up for sorption measurements over the temperature range of (273.15–368.15) K and pressures up to 10 MPa. The main function of this apparatus is to rapidly screen porous materials for the separation of a specific gas mixture by measuring adsorption isotherms. Measurements with empty sample containers were carried out to evaluate potential systematic errors. Validation measurements on commonly studied cases (CO_2_ adsorption on Zeolite 4A, ZIF-8, and Zeolite 13X) were performed, and the excellent agreement with results of literature indicates the high reliability of the measurement system. A comprehensive uncertainty analysis was carried out to determine the uncertainty of measurement results and to propose future improvements of the system. Later, the measurement system was applied to screen suitable materials for the separation of refrigerant blends, which are composed of six pure refrigerants (R-32, R-125, R-1234yf, R-134a, R-1234ze­(E), and R-290). The studied porous materials include six metal–organic framework samples, five zeolite samples, and one activated carbon sample. The results show that all zeolite samples investigated (Köstrolith 3AK, 3ABFK, 3ABFK­(HSD), 4AK, and 4ABFK) exhibit a higher adsorption capacity for R-32 than for the other five refrigerants, with Köstrolith 4ABFK showing the highest selectivity. This makes it possible to use any of these zeolite samples to separate R-32 from the R-410A blend (a mixture of R-32 + R-125, to be phased down in the European Union) or even separate R-32 from the mixtures composed of any of these six refrigerants. The other studied porous samples do not yield any promising results for the refrigerant blend separation.

## Graphical abstract

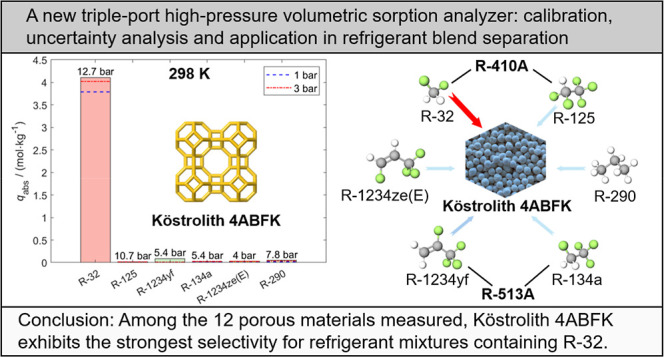

## 1 Introduction

Starting from January 2025, the European Union has implemented a series of more stringent measures under the revised F-gas regulation (Regulation (EU) 2024/573)^1^ aimed at progressively phasing out the use of fluorinated refrigerants with high global warming potentials (GWPs) in specific applications. According to the regulation, the use of fluorinated refrigerants with a GWP of 150 or higher will be prohibited in self-contained commercial refrigerators and freezers, as well as in other self-contained refrigeration equipment (excluding chillers), unless mandated by on-site safety requirements. Similarly, the use of such refrigerants in skin-cooling equipment will be prohibited, except in medical applications. In addition, the revised regulation prohibits the placing on the market of refrigeration systems, excluding chillers and those governed by separate provisions, that contain less than 3 kg of fluorinated refrigerants with a GWP of 750 or higher. As a result, the use of R-410A (GWP: 2088) will be banned in newly manufactured stationary split air conditioning and split heat pump systems. These measures represent a significant step toward the EU’s broader strategy of reducing the environmental impact of high-GWP fluorinated gases, in favor of more sustainable refrigerant alternatives. As environmental standards tighten, the recovery of recyclable pure components with lower GWP values, such as R-32 from R-410A (a 50/50 wt % mixture of R-32 and R-125) and R-1234yf from R-513A (a blend of 44 wt % R-134a and 56 wt % R-1234yf), is expected to become increasingly important. Most refrigerant blends are close-boiling mixtures that often exhibit azeotropic behavior, making traditional separation methods like distillation challenging or impractical. In this scenario, adsorption-based separation techniques offer a promising alternative for effectively separating refrigerant mixtures.^2^

For adsorption-based separation techniques, experimental adsorption isotherm data are essential for selecting a suitable adsorbent for separating of a specific gas mixture. Literature review about adsorption-based separation for refrigerant blends using solid porous materials (metal–organic framework (MOF), zeolite, and activated carbon) is given by Wanigarathna et al.^3^ in 2020 and also by Lin et al.^4^ in 2025. Very few materials are found to be useful for the refrigerant blend separation. Wanigarathna et al.^3,5,6^ demonstrated that Zeolite 4A shows excellent selectivity between R-32 and R-125. Lin et al.^4^ found that ZIF-8 can potentially be used to separate R-32 from R-410A, while ZIF-7 ^7,8^ can separate R-32 from R-410A and R-1234yf from R-513A. To find out more solid porous materials for refrigerant blends separation, a new triple-port high-pressure volumetric sorption analyzer (3HP-VSA) was developed in this work. The key function of this apparatus is to rapidly screen porous materials for the separation of a specific gas mixture by measuring adsorption isotherms. Considering the current popularity and the future trend, six pure refrigerants were investigated: R-32, R-125, R-1234yf, R-134a, R-1234ze­(E), and R-290. Some of their key physical properties are listed in Table 1, mainly including GWP, kinetic diameter, and physical properties. According to the values of kinetic diameter, an adsorbent with a pore size of 4 Å (such as Zeolite 4A) is likely to adsorb R-32, while the adsorption for R-125 is theoretically zero. This supports the findings of Wanigarathna et al.^3,5,6^ The current research is funded by the KETEC (Research Platform Refrigeration and Energy Technology) project^9^ within subproject 3.

**Table 1:** Key Physical Properties of the Studied Refrigerant Gases^a^

				critical point							
R-gases	molecular	GWP	kinetic diameter/Å	*T*_c_/K	*p*_c_/MPa	ρ_c_/kg·m^–3^	molar mass/g·mol^–1^	triple point temperature/K	NBP^b^/K	gas dipole at NBP/Debye	acentric factor
R-290	C_3_H_8_	3	4.3	369.89	4.2512	220.48	44.096	85.525	231.04	0.084	0.1521
R-1234yf	CF_3_CF=CH_2_	4	-	367.85	3.3822	475.55	114.04	122.77	243.67	2.480	0.2760
R-1234ze(E)	CF_3_CH=CFH	6	-	382.51	3.6349	489.24	114.04	169.00	254.18	1.270	0.3130
R-32	CH_2_F_2_	677	3.9	351.26	5.7820	424.00	52.024	136.34	221.50	1.978	0.2769
R-134a	CH_2_FCF_3_	1300	-	374.21	4.0593	511.90	102.03	169.85	247.08	2.058	0.3269
R-125	CHF_2_CF_3_	3170	4.4	339.17	3.6177	573.58	120.02	172.52	225.06	1.563	0.3052

a Most values were obtained from REFPROP 10.0,^10^ while GWP was from,^11^ and kinetic diameter was from.^3,12^

b Normal boiling point.

Inspired by the work of Wanigarathna et al.^3,5,6^ on Zeolite 4A, five zeolite samples (Köstrolith 3AK, 3ABFK, 3ABFK­(HSD), 4AK, and 4ABFK) were chosen for this study. These samples are all commercially available and inexpensive. Furthermore, inspired by the work of Lin et al.,^4^ which reveals that MOF might have higher sorption capacity and outstanding performance, two MOF samples, UiO-66 and MIL-100­(Fe), and their variations were studied. Finally, an activated carbon (Alcarbon UC 50/4 × 8, Donau Carbon GmbH, Germany) was also investigated. The aim of this study is to evaluate the adsorption capacities of these 12 porous materials for six pure refrigerants, and also to identify more potential materials for reclaiming low-GWP components from high-GWP refrigerant mixtures. For example, the reclaim of R-32 from R-410A, R-1234yf from R-513A, and even the selective extraction of reusable R-32, R-1234yf, and R-1234ze­(E) from the mixture of these six refrigerants.

## 2 Research Methods

### 2.1 Apparatus Description

Figure 1 illustrates the schematic of the gas adsorption measurement system based on a 3HP-VSA (type: RuboSORP MPA-3, RUBOLAB, Germany). The core component of the measurement system is the reference cell, which is connected to three high-pressure sample cells (HPSCs), a temperature-measurement cell (TM), two temperature sensors, and three pressure transducers with different measurement ranges (P1:0–10 MPa, P2:0–1 MPa, P3:0–0.15 MPa). The connecting spaces between the reference cell and HPSCs or TM are referred to as the bridge space. This design enables adsorption measurements of three different porous materials within a single experimental cycle. Pressure transducers P2 and P3 are protected by automatic shut-off valves that prevent overpressure damage. Using sensors with different measurement ranges ensures accurate pressure measurements across varying conditions, thereby improving the reliability of the experimental data. The 3HP-VSA features two heating systems: an electric heater capable of heating samples to 573 K within 15 min (from 298 to 573 K) and a circulating bath (Huber Pilot ONE Ministat 240, Peter Huber Kältemaschinenbau AG, Germany) for precise temperature control from 273.15 to 373.15 K. Typically, the electric heater activates the sample, followed by temperature control using the circulating bath. The gas cylinders provide the gas sample to the reference cell. Helium is used for dead volume determination, while refrigerants are employed for adsorption measurements. The classic two-stage vacuum pump (Pfeiffer DUO Line, Pfeiffer Vacuum GmbH, Germany), together with the ventilation system connected to the reference cell, ensures complete removal of the previous gas sample before introducing a new one.

**Figure 1:**
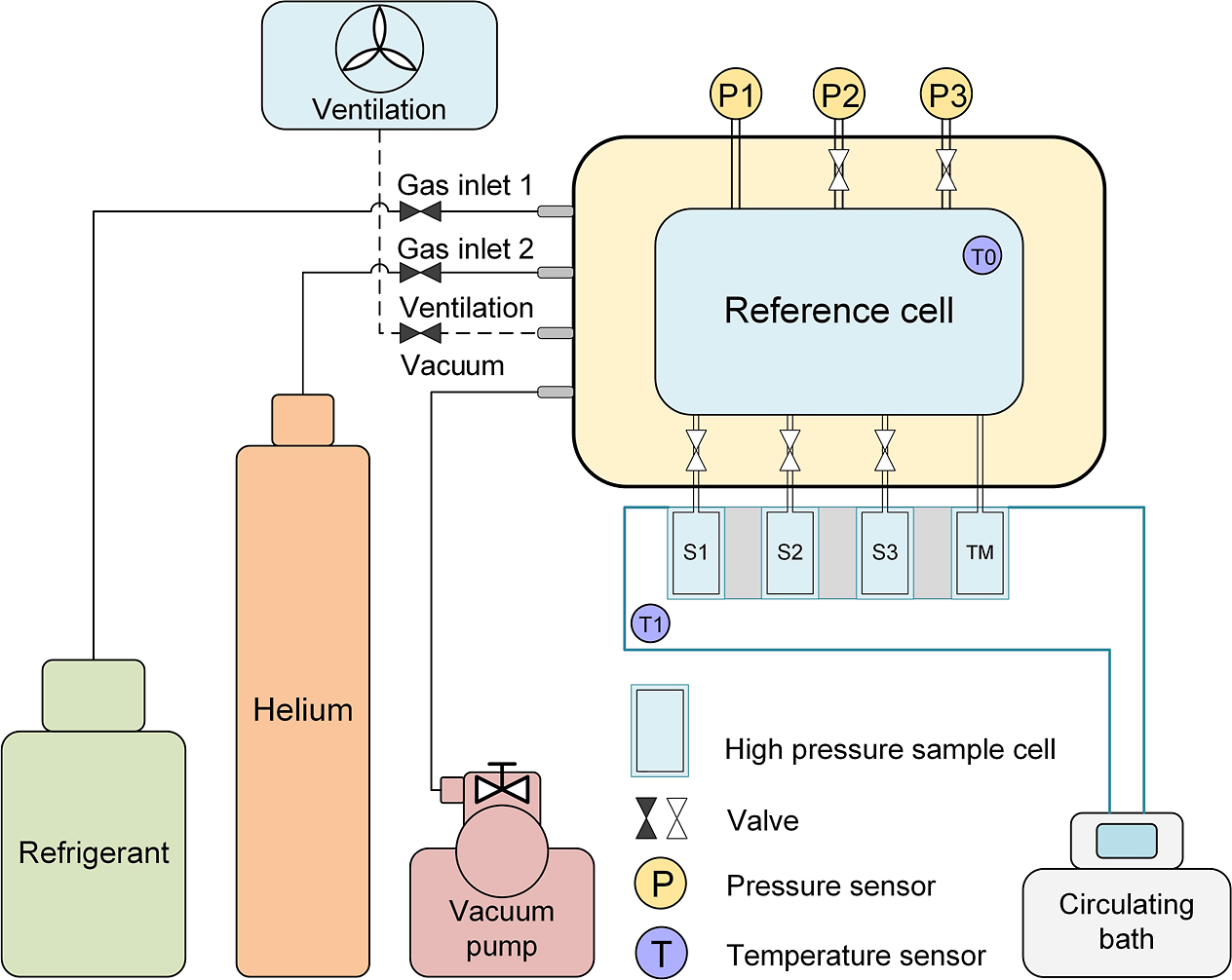
Schematic of the triple-port high pressure volumetric sorption analyzer (3HP-VSA, type: RuboSORP MPA-3, RUBOLAB).

### 2.2 Measurement Principle

In the volumetric (manometric) adsorption technique, a known mass of gas is introduced from a reference cell into a pre-evacuated sample cell containing the adsorbent, and the equilibrium pressure is subsequently recorded once the system stabilizes. The mass of gas adsorbed is determined by calculating the difference between the total mass of gas dosed and the quantity of free gas remaining in the void volume, as inferred from the initial and equilibrium pressures using the equation of state (EoS) of the gas. The fundamental principle underlying the volumetric (manometric) measurement technique is schematically represented in Figure 2. A reference cell with a known volume *V*_0_ is connected via a controllable valve to a sample cell of volume *V*_1_, which contains the activated adsorbent (with a mass weighed in advance with a balance of *W*_s_, and a purity of φ_purity_ (all adsorbent samples are assumed to contain a small fraction of inert impurities that do not contribute to gas adsorption) occupying a volume *V*_s_. The reference and sample cells are maintained at temperatures *T*_0_ and *T*_1_, respectively. Initially, the reference cell is filled with gas to a pressure *p*_0_, while the sample cell is evacuated. The total mass of the gas initially contained in the reference cell, *m*_0_, is calculated using the expression

1 $$m_{0}=\rho_{g}\left(T_{0},p_{0}\right)\cdot V_{0}$$

where ρ_g_(*T*_0_, *p*_0_) represents the gas density at the initial temperature *T*_0_ and pressure *p*_0_. The density can be calculated with a reference EoS implemented in REFPROP 10.0.^10^

**Figure 2:**
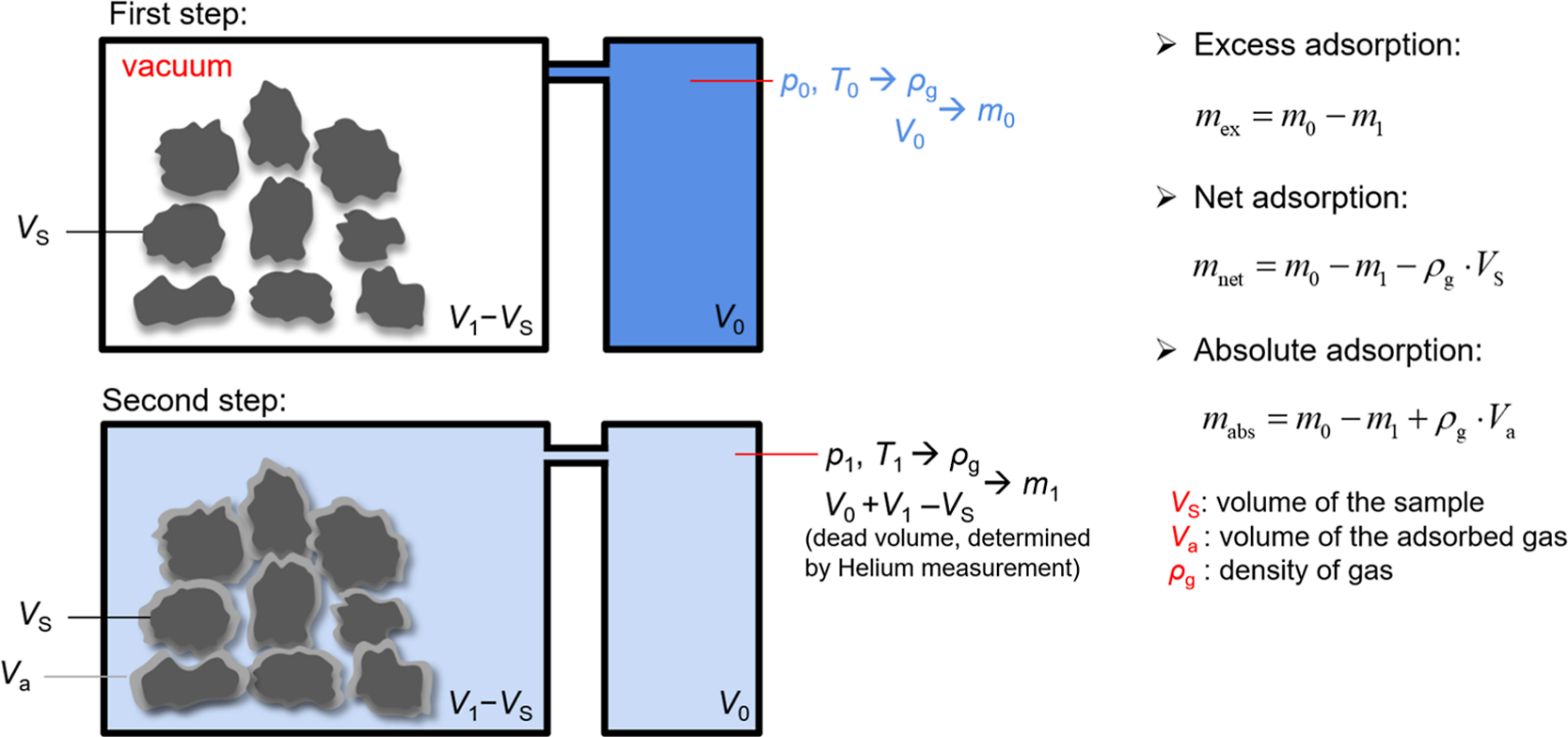
Schematic of the adsorption measuring principle of a volumetric technique.

After valve opening, adsorption equilibrium is determined by a pressure change below a preset threshold within a defined time interval. At this point, the following mass *m*_1_ can be calculated

2 $$m_{1}=\rho_{g}\left(T_{0},p_{1}\right)\cdot V_{0}+\rho_{g}\left(T_{1},p_{1}\right)\cdot \left({V_{Dead}-V}_{0}\right)$$

where ρ_g_(*T*_1_,*p*_1_) denotes the gas density at equilibrium temperature *T*_1_ and pressure *p*_1_. Here, *V*_Dead_ = *V*_0_ + *V*_1_ – *V*_s_ is commonly referred to as the dead volume (as shown in Figure 2), which can be experimentally determined using an inert, nonadsorbing gas such as helium. Please note, for our system, volumes of pressure sensor 2, *V*_p2_, pressure sensor 3, *V*_p3_, and the bridge space *V*_pb_, should also be considered. Considering the mass balance of the system, the following expression holds

3 $$m_{0}=m_{1}+m_{abs}-\rho_{g}\left(T_{1},p_{1}\right)\cdot V_{sorp}$$

here, *V*_sorp_ is the volume of the adsorbate. Combining the relationship between absolute and excess adsorption

4 $$m_{ex}=m_{abs}-\rho_{g}\left(T_{1},p_{1}\right)\cdot V_{sorp}$$

the excess adsorption is then given by

5 $${m_{ex}=m}_{0}-m_{1}$$

To obtain a complete adsorption isotherm, the pressure gradually increases throughout the measurement process. At each pressure step, the pressure in the reference cell was maintained higher than that in the sample cell prior to opening the connecting valve. At step *n* – 1, the total mass *m*_t,*n*–1_ in the system is

6 $$m_{t,n-1}=m_{R,n-1}+m_{S,n-1}+m_{abs,n-1}$$

*m*_R,*n*–1_ is the mass of the gas in reference cell, *m*_S,n–1_ and *m*_abs,*n*–1_ are the masses of gas that is in sample cell and the mass of gas that has been absorbed. After opening the valve and reaching equilibrium, the total mass *m*_t,*n*_ in the system is

7 $$m_{t,n}=m_{R,n}+m_{S,n}+m_{abs,n}$$

When the mass constants are considered, it follows that *m*_t,*n*–1_ = *m*_t,*n*_ = const. The adsorbed gas amount *m*_abs,*n*_ can be described using eqs 6 and 7

8 $$m_{abs,n}=m_{R,n-1}+m_{S,n-1}+m_{abs,n-1}-m_{R,n}-m_{S,n}$$

The sorption capacity *q* of the adsorbent sample can then be calculated as

9 $$q_{i}=\left(m_{i}/M_{gas}\right)/m_{sample},\left(i=ex,\text{or}\;abs\right)$$

where *M*_gas_ is the molar mass of the gas and *m*_sample_ is the mass of the adsorbent sample.

### 2.3 Experimental Procedures

Prior to each adsorption isotherm measurement, the mass of the sample *m*_sample_ was determined. The samples were then activated in evacuated HPSCs at 423 (±5) K and at pressure below 1.0 Pa. After activation, the samples were no longer exposed to air. However, since it is not feasible to directly measure the sample mass after activation, a φ_activate_ = 2.0% mass loss is assumed with an expanded (*k* = 2) uncertainty of 1.0%, i.e., the actual mass loss lies within the range of 1.0–3.0% with a confidence level of approximately 95%. In this context, the effective sample mass is

10 $$m_{sample}=W_{s}\cdot \varphi_{purity}\cdot \left(1-\varphi_{activate}\right)$$

This estimated mass loss is primarily due to the removal of adsorbed water and other gaseous impurities originally present in the sample. The 2.0% value is estimated based on experiments with Zeolite 4A, Zeolite 4ABFK, ZIF-8, and Zeolite 13X, see Table S2 in the Supporting Information, in which the sample mass before activation was compared with the mass measured after activation and brief exposure to air.

When changing to a new gas, the activation time for the first isotherm lasted for 24 h, and for the remaining isotherms, the sample was reactivated for 8 h. After activation, the temperature control system was set to the designated temperature (283, 298, 303, or 313 K). Once the temperature stabilized (fluctuation at 0.05 K in 1 min), isothermal measurement could start with a pressure-increasing step. The experiment was fully automated, requiring parameter setup via a computer interface, with data acquisition and storage managed automatically. The highest measurement pressure is generally that at 0.1 MPa below the saturation pressure. For example, at 303 K, the saturation pressures are 1.2 MPa for R-32,^13^ 1.0 MPa for R-125,^14^ 0.5 MPa for R-1234yf,^15^ 0.45 MPa for R-134a,^16^ 0.4 MPa for R-1234ze­(E),^17^ and 0.7 MPa for R-290.^18^ For each adsorption measurement, before any refrigerant gases were introduced, a helium measurement was performed to determine the dead volume by assuming that helium is not adsorbed on any porous material.

### 2.4 Porous Materials and Gas Samples

In this work, the measurement system was applied to measure adsorption isotherms of six pure refrigerants (R-32, R-125, R-1234yf, R-134a, R-1234ze­(E), and R-290) on various porous samples. The studied porous materials (as shown in Table 2) include six zeolite samples (Köstrolith 3AK, 3ABFK, 3ABFK­(HSD), 4AK, 4ABFK, and 13X), seven metal–organic-framework samples (UiO-66, AI-UiO-66, DETA-UiO-66, Fe@MIL-100­(Fe), Ca@MIL-100­(Fe), Mg@MIL-100­(Fe), and ZIF-8), and one activated carbon sample (Alcarbon UC 50/4 × 8). The zeolites were provided by CWK (Chemiewerk Bad Köstritz GmbH, Germany), and activated carbon was supplied by Donau Carbon (Donau Carbon GmbH, Germany). These are standard commercial materials; therefore, they were used as received, without any further processing or characterization. The synthesis procedures and characterization details of the three UiO-66 samples and the three MIL-100 samples were described in detail in refs 19–21 Note that, in the original publications, the three MIL samples were denoted as MIL-100 (Fe^3+^, Fe^2+^), MIL-100 (Fe^3+^, Ca^2+^), and MIL-100 (Fe^3+^, Mg^2+^); whereas in the present study, they are referred to as Fe@MIL-100­(Fe), Ca@MIL-100­(Fe), and Mg@MIL-100­(Fe), respectively. In addition, a ZIF-8 sample was used to validate the measurement system. It was synthesized using the method proposed by Zhang et al.^22^ and characterized with XRD tests at the Materials Center of Dresden University of Technology (TUD).

**Table 2:** Summary of Sample Information

sample name	supplier	BET area (m^2^/g)
UiO-66	Tianjin University	850 ^23^
Al-UiO-66	Tianjin University	1106 ^23^
DETA-UiO-66	Tianjin University	386 ^21^
Fe@MIL-100(Fe)	Tianjin University	1420 ^19^
Ca@MIL-100(Fe)	Tianjin University	1600 ^19^
Mg@MIL-100(Fe)	Tianjin University	1860 ^19^
ZIF-8	Dresden University of Technology	1168^a^
Köstrolith 3AK	CWK Chemiewerk Bad Köstritz GmbH	25^b^
Köstrolith 3ABFK	CWK Chemiewerk Bad Köstritz GmbH	25^b^
Köstrolith 3ABFK (HSD)	CWK Chemiewerk Bad Köstritz GmbH	25^b^
Köstrolith 4AK (Zeolite 4A)	CWK Chemiewerk Bad Köstritz GmbH	31^c^
Köstrolith 4ABFK	CWK Chemiewerk Bad Köstritz GmbH	31^c^
Köstrolith 13X (Zeolite 13X)	CWK Chemiewerk Bad Köstritz GmbH	730^a^
Alcarbon UC 50/4 × 8	Donau Carbon GmbH	958^d^

a Values provided by the supplier.

b The BET value for zeolite 3A^24–26^ was taken from the literature, determined from N2 adsorption isotherms at 77. However, the sample supplier recommends using CO2 or Ar adsorption isotherms for more reliable results. Due to experimental limitations, such measurements could not be performed in the present study.

c The BET value for zeolite 4A^27,28^ was taken from the literature, determined from N2 adsorption isotherms at 77. However, the sample supplier recommends using CO2 or Ar adsorption isotherms for more reliable results. Due to experimental limitations, such measurements could not be performed in the present study.

d Determined with 77 K adsorption measurement with N_2_ in this work.

Gas samples of R-32, R-125, R-1234yf, and R-290 (purity 0.995) were supplied by TEGA, and those of R-134a and R-1234ze­(E) (purity 0.995) were from Westfalen. CO_2_, N_2_, and Ar, with purities of 0.99995, 0.999999, and 0.999999, respectively, were used for validation measurements and provided by Air Liquide. Helium (purity 0.999999) was supplied by Nippon Gases. Specific information about the gases used in this study is given in Table S1 in the Supporting Information.

### 2.5 Isotherm Model

The measured sorption isothermal data were fitted to the Langmuir isothermal model^29^

11 $$q_{Lang}=q_{m}\cdot \frac{K_{0}\cdot \exp\left(-\frac{\Delta H}{R\cdot T}\right)\cdot p}{\left[1+K_{0}\cdot \exp\left(-\frac{\Delta H}{R\cdot T}\right)\cdot p\right]}$$

where *q*_Lang_ is the adsorption capacity at pressure *p* and temperature *T*, *q*_m_ is the maximal adsorption capacity, *K*_0_ is the gas–solid affinity coefficient, Δ*H* is the enthalpy of adsorption, *R* is the universal gas constant.

## 3 Commissioning of the New Measurement System

The commissioning of the new measurement system involved: measurements with empty sample cells to evaluate the systematic errors, validation measurements on commonly studied cases with comparison to literature, and based on these, a comprehensive uncertainty analysis to determine the measurement uncertainty and to propose future improvements of the system.

### 3.1 Measurements with Empty Measurement Cells

Prior to conducting adsorption measurements on porous materials, experiments with empty measurement cells (hereafter referred to as empty measurements) were performed to evaluate the systematic errors of the apparatus. A dummy sample mass of 6 g was entered into the apparatus’s control software to enable the experiment to proceed. Theoretically, in the absence of a porous material, the absolute uptake *q*_abs_ should be zero. However, empty measurements using three gases (N_2_, CO_2_, and Ar) revealed deviations from zero. The empty measurements for N_2_ are shown in Figure 3 and the corresponding results for CO_2_ and Ar are provided in Figures S1 and S2 in the Supporting Information. At 283 K, the adsorption of N_2_ was negative, with an error of approximately 0.3 mmol (corresponding to 0.05 mmol·g^–1^ × 6 g) at 1.0 MPa. At 298 and 303 K, the results were positive, with an error of approximately 0.012 mmol (corresponding to 0.002 mmol·g^–1^ × 6 g) at 1.0 MPa. The same trend was also observed for CO_2_ and Ar. Moreover, the deviation increased with rising pressure, primarily because the uncertainties related to gas mass determination accumulate (see eq 8) during the isothermal measurements.

**Figure 3:**
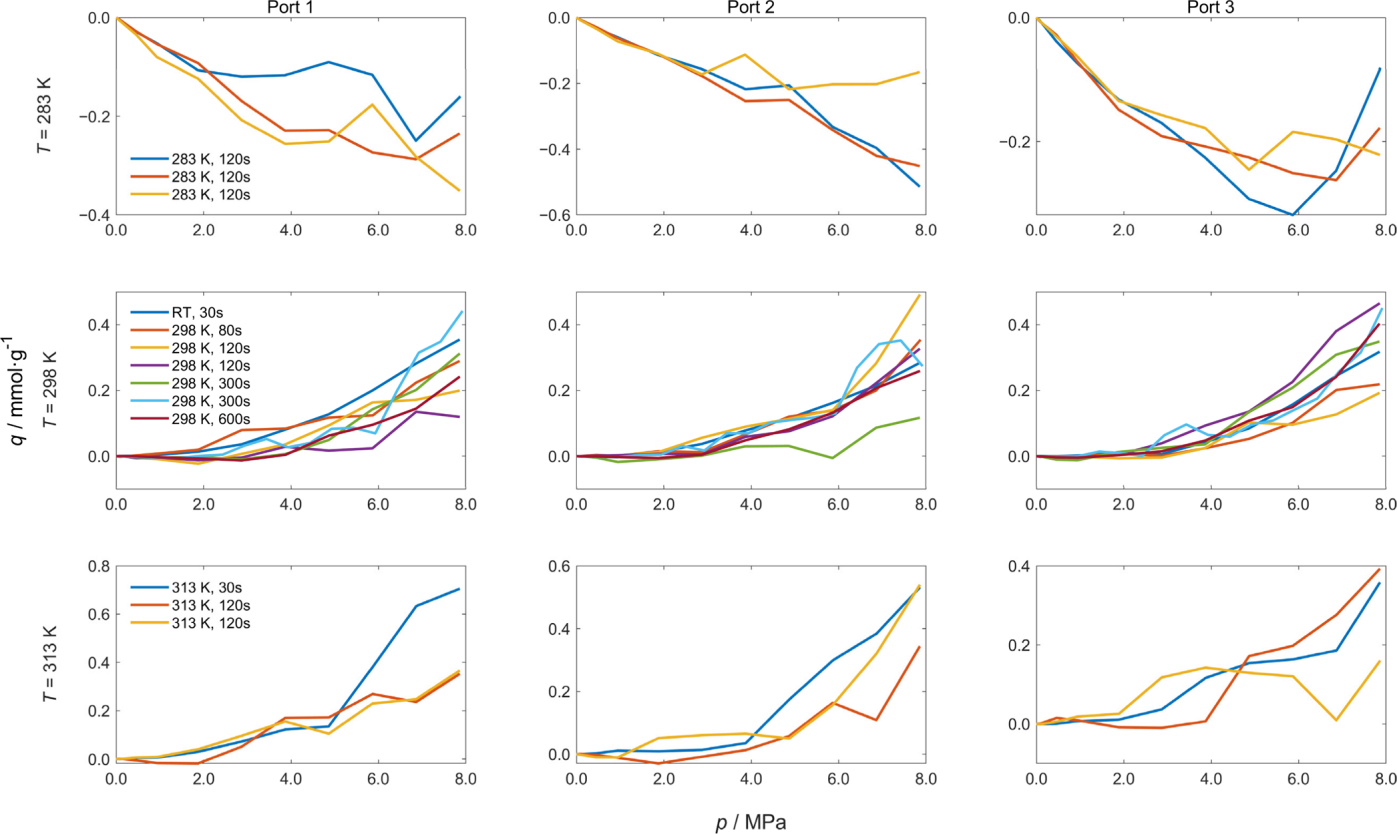
Isothermal adsorption curves of N_2_ obtained from empty measurements, assuming a nominal sample mass of 6 g for reference purposes.

### 3.2 Validation Measurements

In the beginning, a few “repeated” measurements were carried out for self-validation. The word “repeated” is quoted because the measurements were not repeated exactly with the same set of conditions, but with one different condition. The self-validation tests were carried out for the adsorption of CO_2_ on Zeolite 4A at 283 K up to 4.4 MPa, with the sample mass approximately 4.1, 2.5, and 0.0 g in each port. Here, the different condition is the masses. In addition, for all adsorption measurement systems employing volumetric techniques, the equilibrium condition is a critical operational parameter. The equilibrium condition is reached if the pressure change is less than a given pressure threshold within a given time interval. Only upon reaching equilibrium can the system reliably acquire data for analysis and subsequently advance to the next pressure point. In this work, different equilibrium condition settings were tested with three-time intervals: 40, 60, and 80 s, but with the same pressure threshold, 1.0 kPa, which is of the same order as the pressure uncertainty. The results, as given in Figures S3 and S4 in the Supporting Information, demonstrate that in the low and medium pressure range (*p* ≤ 4.0 MPa), the repeatability is excellent, i.e., the measured *q*_abs_(*p*) is independent of the sample mass and unaffected by the time intervals required to reach equilibrium. However, the high relative deviation at 5.0 MPa (up to 8.0%) implies that the measurement system has relatively high uncertainty in the high-pressure range. Please note, we knew in advance that the adsorption kinetics of CO_2_ on Zeolite 4A is fast; for slow adsorption kinetics, the time intervals should be set longer when necessary.

Following the completion of the apparatus’s self-validation process, additional comparative experiments were conducted on well-studied cases of CO_2_ adsorption on ZIF-8, Zeolite 4A, and Zeolite 13X to benchmark the results against previously published studies. The adsorption isotherms of CO_2_ on these materials at three temperatures (283, 298, and 313 K) were presented in Figures 4–6, respectively, with detailed test data provided in the Supporting Information.

**Figure 4:**
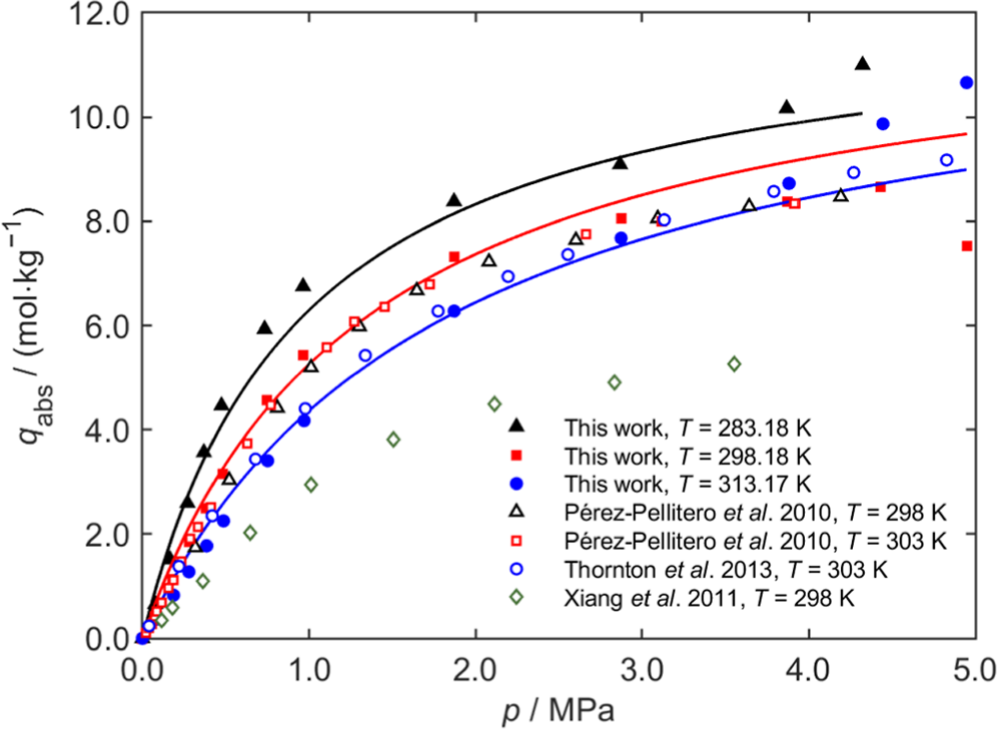
CO_2_ adsorption curves on ZIF-8 at different temperatures. The solid curves are the fits of the Langmuir model to the experimental data of this work.

**Figure 5:**
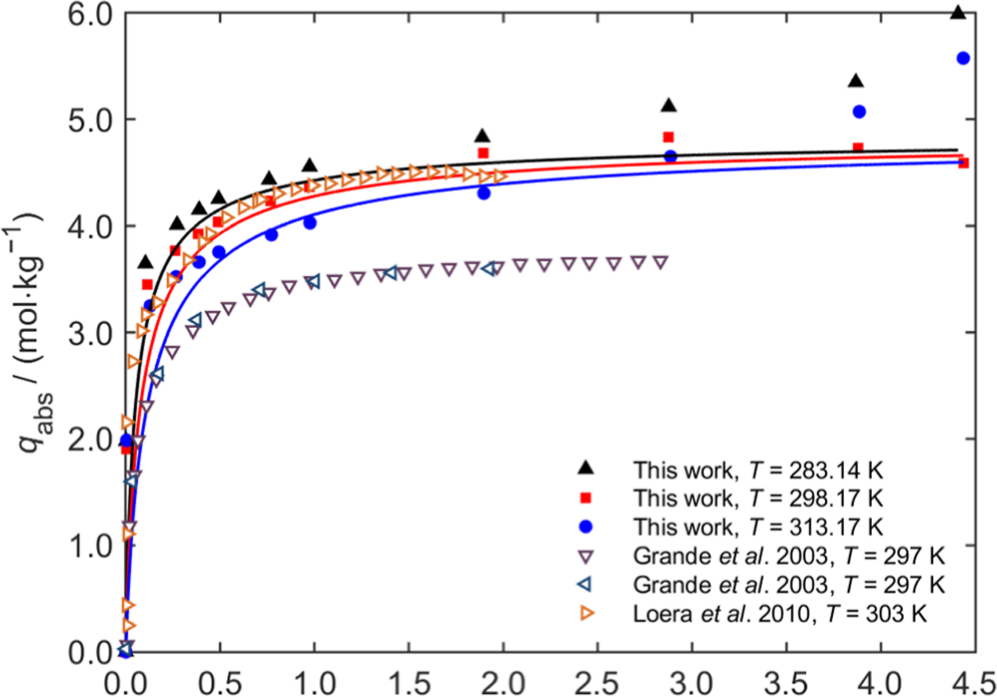
CO_2_ adsorption curves on Zeolite 4A at different temperatures. The solid curves are the fits of the Langmuir model to the experimental data of this work.

**Figure 6:**
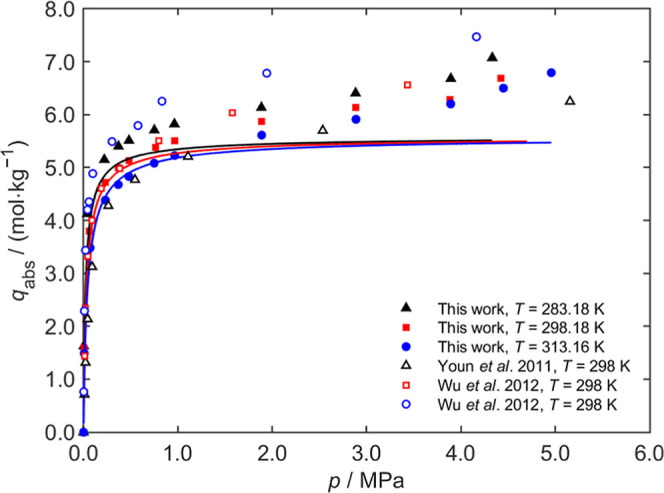
CO_2_ adsorption curves on Zeolite 13X at different temperatures. The solid curves are the fits of the Langmuir model to the experimental data of this work.

Table 3 shows the Langmuir parameters (*q*_m_, *K*_0_ and Δ*H*) of CO_2_ on three materials (ZIF-8, Zeolite 4A, and Zeolite 13X) and their standard statistical uncertainties, *u*, together with the root mean square deviation (RMSD) of the fit. For parameters with strong physical significance (*q*_m_ and Δ*H*), the fitted uncertainties *u* are smaller than the corresponding fitted values for ZIF-8, indicating both a successful fit and reliable parameter estimation for this material. In contrast, for Zeolite 4A and Zeolite 13X, the uncertainty of Δ*H* exceeds 150% of the fitted value and attempts to apply the Toth model^30^ did not improve the fit. This suggests that CO_2_ adsorption on Zeolite 4A and Zeolite 13X cannot be adequately represented by simple isotherm models such as Langmuir or Toth.

**Table 3:** Langmuir Parameters of CO_2_ on Three Materials (ZIF-8, Zeolite 4A, and Zeolite 13X) and Their Standard Statistical Uncertainties, *u*, Together with the Root Mean Square Deviation of the Fit

	ZIF-8		Zeolite 4A		Zeolite 13X	
parameters	value	*u*	value	*u*	value	*u*
*q*_m_ (mol·kg^–1^)	12.2820	0.8246	4.8472	0.6536	5.5988	0.4072
10^5^ *K* (MPa^–1^)	124.5300	163.7900	653.8600	7784.2000	347.9000	2403.4000
Δ*H* (kJ·mol^–1^)	–15.8660	3.2202	–19.5310	30.7990	–23.4240	17.2480
RMSD (mol·kg^–1^)	0.3026		0.6166		0.3416	

Figure 4 presents the adsorption data for ZIF-8, including fitted curves and comparisons with literature data. In general, the adsorption capacity increases with pressure and eventually approaches a plateau. At the same pressure, the adsorption capacity typically decreases with increasing temperature. The adsorption isotherms were well fitted by the Langmuir model. Our experimental results are very close to those of Pérez-Pellitero et al.^31^ and Thornton et al.,^32^ and higher than those reported by Xiang et al.^33^ Figure 5 shows comparisons with literature data from Grande et al.^34^ and Loera et al.,^35^ indicating that the CO_2_ adsorption data for Zeolite 4A at 298 and 303 K were consistent with reported trends and values. At pressures exceeding 4 MPa, deviations were observed between the experimental data and the fitted curves. These discrepancies were attributed to experimental uncertainties at high pressures, limitations of the fitted models, or phenomena such as capillary condensation under high-pressure conditions. Data above 4 MPa were excluded from fitting to the Langmuir model in this study to avoid misrepresentation. In Figure 6, the adsorption isotherms of Zeolite 13X were compared with the data from Youn et al.^36^ and Wu et al.^37^ The trends observed in the measurements were consistent with those reported in the literature, showing similar adsorption behavior. However, the adsorption values of this study were slightly lower, which could have been due to the activation temperature used for Zeolite 13X in this study (423 K), whereas the recommended activation temperature is 573 K. This difference in activation temperature may have affected the surface area and availability of adsorption sites.

### 3.3 Uncertainty Analysis

In this work, measurement uncertainties were estimated in accordance with the “Guide to the Expression of Uncertainty in Measurement”^38^ and the sensitivity method proposed by Bernardini et al.^39^ Unless otherwise stated, the uncertainties mentioned in this work are all expanded uncertainties with a coverage factor of 2 (*k* = 2), which corresponds to a confidence level of 95.4%. Uncertainties associated with measurements and with impurities in the porous material samples were taken into account, whereas those associated with the impurities in the gas samples (negligibly small because of the high gas purity) were not.

For sorption capacity *q*, according to eqs 1–10, the uncertainty sources include φ_purity_, *W*_s_, *M*_gas_, φ_activate_, *T*_0_, *T*_1_, *p*, *V*_Dead_, *V*_0_, *V*_p2_, *V*_p3_, *V*_pb_, ρ_sorb_, and ρ_g_. φ_purity_ represents the purity of the 14 adsorbent samples, and each of them has different amounts of impurities. An average purity value of 98% was assumed for all the studied samples with an uncertainty of 2% (i.e., the purities are assumed to be in the range 96 to 100%). *W*_s_ is the weight of adsorbent, measured with an external analytical microbalance (Sartorius Lab Instruments GmbH & Co. KG, Germany, type: MCE224S-2S00–U). Considering the effects of air buoyancy and the sensitivity of the balance, the typical uncertainty associated with the weighing process was approximately 200 μg. *M*_gas_ is the molar mass of the adsorbed gas, the uncertainty of molar mass values for gases can arise from isotopic composition variations, instrumental limitations during measurements, and compositional uncertainties in gas mixtures. For pure gases, these uncertainties are typically negligible but are considered in this work. *T*_0_ and *T*_1_ are the temperatures in the reference cell and sample cell, respectively. Since the temperature sensors were calibrated only by the sensor provider and there could be a large temperature gradient in the large cell, the uncertainty in the temperature measurement was estimated to be 0.2 K. Pressure measurements have uncertainties of 0.05% of the full scale (as specified by the supplier: KELLER Druckmesstechnik AG). *V*_Dead_ refers to the dead volume, which is the volume measured during volume calibration using helium. Its relative uncertainty is estimated to be 1.0%. *V*_0_, *V*_p2_, *V*_p3_, and *V*_pb_ are the volumes of the reference cell, pressure sensor 2, pressure sensor 3, and the bridge spaces, respectively. Their relative uncertainties were estimated to be 0.5%. ρ_sorb_ is the density of the adsorbate, whose value was estimated as the saturated liquid density at the measured temperature with a relative uncertainty of 10%.^40,41^ The gas density ρ_g_ is calculated based on pressure, temperature, and the reference EoS. According to the used reference EoS, the relative uncertainty of ρ_g_ is estimated to be 0.03% for CO_2_ ^42^ and R-290.^18^ 0.05% for R-32 ^13^ and R-134a.^16^ 0.1% for R-125,^14^ R-1234yf,^15^ and R-1234ze­(E).^17^

Typically, the combined expanded uncertainty (*k* = 2) of the sorption capacity, *U*_C_(*q*), was estimated by the error propagation principle^38^

12 $${U_{C}\left(q\right)}^{2}=\sum_{i}{\left(U_{X_{i}}\left(q\right)\right)}^{2}$$

where *X*_*i*_ represents the individual uncertainty contributor and *U*_*X*i_(*q*) refers to uncertainty contribution to *q* only attributed to the contributor *X*_*i*_. *U*_*X*_*i*__(*q*) is estimated according to the sensitivity method^39^ as

13 $$U_{X_{i}}\left(q\right)=\max\left(\left|q\left(X_{i}+\Delta X_{i},X_{j}\right)-q\left(X_{i},X_{j}\right)\right|,\left|q\left(X_{i}-\Delta X_{i},X_{j}\right)-q\left(X_{i},X_{j}\right)\right|\right)$$

here *X*_*j*_ represents all other variables that are fixed in the calculation.

Owing to the underlying experimental principles, several uncertainty components, i.e., those associated with temperature, pressure, volume determination, and density calculation (*T*_0_, *T*_1_, *p*, *V*_Dead_, *V*_0_, *V*_p2_, *V*_p3_, *V*_pb_, ρ_g_), are cumulative in nature; while others (φ_purity_, *W*_s_, *M*_gas_, φ_activate_, ρ_sorb_) are not. As a result, these uncertainties propagate through successive pressure points during data analysis, leading to an amplification of the overall measurement uncertainty. Therefore, the combined uncertainty in the present study is expressed as follows

14 $${\left(U_{C}\left(q_{k}\right)\right)}^{2}=\sum_{i\epsilon I_{non‐acc}}{\left(U_{X_{i}}\left(q_{k}\right)\right)}^{2}+\sum_{k=1}^{n}\sum_{i\epsilon I_{acc}}{\left(U_{X_{i}}\left(q_{k}\right)\right)}^{2}$$

here, *n* is the index of the pressure point, *I*_non‑acc_ refers to the set of nonaccumulated uncertainty sources including φ_purity_, *W*_s_, *M*_gas_, φ_activate_, ρ_sorb_, and *I*_acc_ refers to set of accumulated uncertainty sources, including *T*_0_, *T*_1_, *p*, *V*_Dead_, *V*_0_, *V*_p2_, *V*_p3_, *V*_pb_, ρ_g_.

Here we take the measurements (as shown in Table 4) of CO_2_ adsorption on ZIF-8 at *T* = 283.2 K at one low- and one high-pressure point (*p* = 0.0775 and 4.322 MPa) as illustrative examples. The 14 sources of uncertainty are detailed in Table 4. The analysis indicates that at low pressure (0.0775 MPa, *U*_C_(*q*) = 0.0432 mmol/g, *U*_C_(*q*)/*q* = 6.404%), the dominant sources of uncertainty are associated with pressure measurement and sample purity. In contrast, at high pressure (4.332 MPa, *U*_C_(*q*) = 0.1172 mmol/g, *U*_C_(*q*)/*q* = 1.026%), apart from the cumulative contributions, the dead volume determination becomes the most significant contributor. These findings offer valuable insight into the prioritization of uncertainty reduction strategies: efforts to minimize measurement uncertainty in the future should primarily focus on the above-mentioned three sources, rather than on secondary or less influential factors.

**Table 4:** Uncertainty Budget Table for Sorption Capacity *q* of CO_2_ on ZIF-8 at Two Pressure Points (0.0775 and 4.322 MPa) at *T* = 283.2 K

		contribution to *U*_C_(*q*)/*q*	
uncertainty sources (*x*_*i*_)	uncertainty *U*(*x*_*i*_)	*q* = 0.6746 mmol/g	*q* = 11.4250 mmol/g
adsorbent sample purity φ_purity_ (98%)^a^	2.0%	0.0208	0.0208
weight of adsorbent *W*_s_ (1.4411 g)^a^	0.0002 g	0.0001	0.0001
molar mass of the gas *M*_gas_ (44.0098 g/mol)^a^	0.0020 g/mol	0.0000	0.0000
activation weight loss φ_activate_ (2%)^a^	1.0%	0.0103	0.0103
temperature in reference cell *T*_0_ (298.15 K)	0.20 K	0.0011	0.0090
temperature in sample cell *T*_1_ (283.19 K)	0.20 K	0.0004	0.0094
pressure measurements (*p*) (0.0775 or 4.322 MPa)	0.05% f.s.	0.0339	0.0238
dead volume (*V*_Dead_) (21.579 cm^3^)	0.01·*V*_Dead_	0.0104	0.0598
volume of reference cell *V*_0_ (174.6 cm^3^)	0.005·*V*_0_	0.0087	0.0254
volume of pressure sensor 2 (*V*_p2_) (8.6569 cm^3^)	0.005·*V*_p1_	0.0000	0.0000
volume of pressure sensor 3 (*V*_p3_) (8.0551 cm^3^)	0.005·*V*_p2_	0.0000	0.0000
volume of bridge space (*V*_pb_) (5.50 cm^3^)	0.005·*V*_pb_	0.0000	0.0009
density of the adsorbate (ρ_sorb_)(996 kg/m^3^)^a^	0.1·ρ_sorp_	0.0001	0.0128
density calculation (ρ_g_) (1.4551 or 124.42 kg/m^3^)	0.0003·ρ_g_	0.0010	0.0012
accumulation from previous pressure point		0.0000	0.1084
relative combined expanded uncertainty (*k* = 2) in sorption capacity *U*_C_(*q*)/*q*		**0.0432**	**0.1172**

a Sources that are not accumulated.

## 4 Results and Discussion

This chapter presents the adsorption behavior of six refrigerants on 12 porous materials, with the adsorption data fitted using the Langmuir model. Comparisons are made between the model-predicted adsorption capacities at 1 bar, 3 bar, and the highest measured adsorption pressure. The results provide guidance on the potential application of these 12 porous materials for the separation of mixed refrigerants. All adsorption data are provided in the Adsorption Information File (AIF) format^43^ and as plain-text files including uncertainty information, in a ZIP archive in the Supporting Information.

### 4.1 Zeolite Samples

For adsorption of six refrigerants on five zeolites, all samples exhibit high absolute adsorption capacities (*q*_abs_) for R-32 (ranging from 2.1 mol·kg^–1^ to 4.1 mol·kg^–1^) and significantly lower *q*_abs_ values for the other five refrigerants (ranging from 0.015 mol·kg^–1^ to 0.085 mol·kg^–1^). Here, we have selected the two best-performing zeolite materials (Köstrolith 4A and 4ABFK) for detailed discussion. Sorption measurements of R-32 and R-125 on Köstrolith 4AK and 4ABFK at three isotherms (283, 298, and 313 K) and pressures up to 1.27 and 0.50 MPa are depicted in Figures 7 and 8, respectively. To reduce the size of the main manuscript, the remaining adsorption isotherms of six refrigerant gases (R-32, R-125, R-1234yf, R-134a, R-1234ze­(E), and R-290) on these five zeolite materials (Köstrolith 3AK, 3ABFK, 3ABFK­(HSD), 4AK, and 4ABFK) are presented in Figures S5 to S16 in Supporting Information, respectively. As shown in Figures 7 and 8, when the pressure increases, the *q*_abs_ of R-32 on the zeolite materials increase rapidly at low pressure and then reach a plateau. At the same pressure, *q*_abs_ decreases as the temperature increases. The fitted Langmuir model curves are plotted in each figure, with the corresponding fitted parameters provided in Tables 5 and 6, along with the standard statistical uncertainties (*u*) and the RMSD of the fit. The RMSD is generally less than 5% of the maximal sorption capacity of the isotherms. For parameters with strong physical significance (*q*_m_ and Δ*H*), the fitted uncertainties *u* are generally smaller than the corresponding fitted values, except for Köstrolith 4ABFK with R-125. Applying the Toth model^30^ did not improve this exceptional case, primarily due to the very low adsorption capacity combined with relatively high measurement uncertainty. The Langmuir fitting parameters for the six refrigerants on five zeolite materials can be found in Tables S3 to S14 in the Supporting Information.

**Figure 7:**
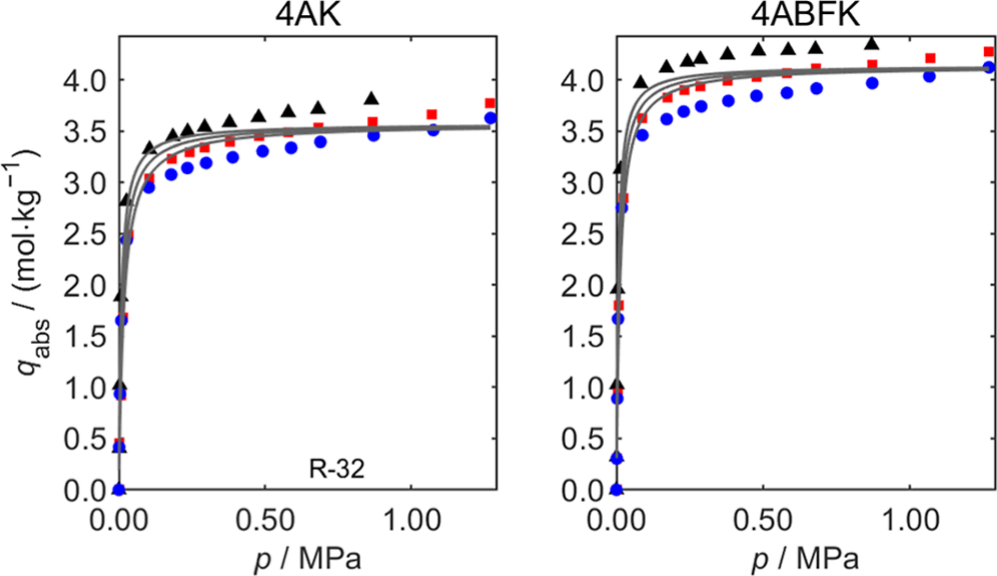
Absolute sorption capacity *q*_abs_ of the pure refrigerant R-32 on Köstrolith 4AK and 4ABFK at different pressures *p*. (black ▲), *T* = 283 K; (red ■), *T* = 298 K; (blue ●), *T* = 313 K. Pressure range: 0–1.27 MPa. The solid curves are the fits of the Langmuir model to the experimental data.

**Figure 8:**
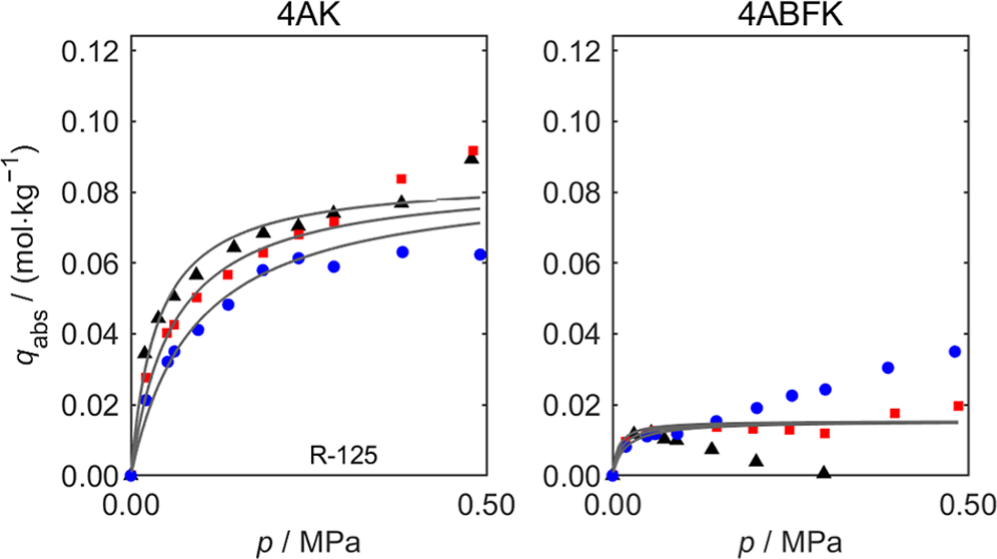
Absolute sorption capacity *q*_abs_ of the pure refrigerant R-125 on Köstrolith 4AK and 4ABFK at different pressures *p*. (black ▲), *T* = 283 K; (red ■), *T* = 298 K; (blue ●), *T* = 313 K. Pressure range: 0–0.50 MPa. The solid curves are the fits of the Langmuir model to the experimental data.

**Table 5:** Langmuir Parameters of R-32 and R-125 on Köstrolith 4AK and Their Standard Statistical Uncertainties, *u*, Together with the Root Mean Square Deviation of the Fit

	R-32		R-125	
parameters	value	*u*	value	*u*
*q*_m_ (mol·kg^–1^)	3.574	0.143	0.084	0.006
10^5^ *K* (MPa^–1^)	8432	29,819	153.61	426.81
Δ*H* (kJ·mol^–1^)	–16.997	8.737	–23.131	6.968
RMSD (mol·kg^–1^)	0.173		0.004	

**Table 6:** Langmuir Parameters of R-32 and R-125 on Köstrolith 4ABFK and Their Standard Statistical Uncertainties, *u*, Together with the Root Mean Square Deviation of the Fit

	R-32		R-125	
parameters	value	*u*	value	*u*
*q*_m_ (mol·kg^–1^)	4.147	0.157	0.015	0.008
10^5^ *K* (MPa^–1^)	7901	2758	2558.7	112,960
Δ*H* (kJ·mol^–1^)	–23.581	8.615	–20.102	112.09
RMSD (mol·kg^–1^)	0.191		0.007	

To provide a more intuitive comparison of the zeolite materials for the refrigerant gases, Figure 9 presents the absolute adsorption values *q*_abs_ of the five zeolite materials for the six refrigerant gases at three pressures (1 bar, 3 bar, and the highest measured pressure). In addition, Figure 10 shows the calculated ratio of the adsorption values for R-32 and R-125 on the five zeolite materials at the same pressure. As illustrated in Figure 9, all five zeolites exhibit notably higher adsorption capacities for R-32 compared to the other refrigerant gases. Among them, Köstrolith 4ABFK shows superior performance, with adsorption ratios for R-32 and R-125 consistently exceeding 270 at 1 bar, 3 bar, and the highest measured pressure. This suggests that these materials have the potential to separate R-32 from R-410A. Furthermore, it is also feasible to use these zeolite materials to separate R-32 from its mixture with any of the other five refrigerants.

**Figure 9:**
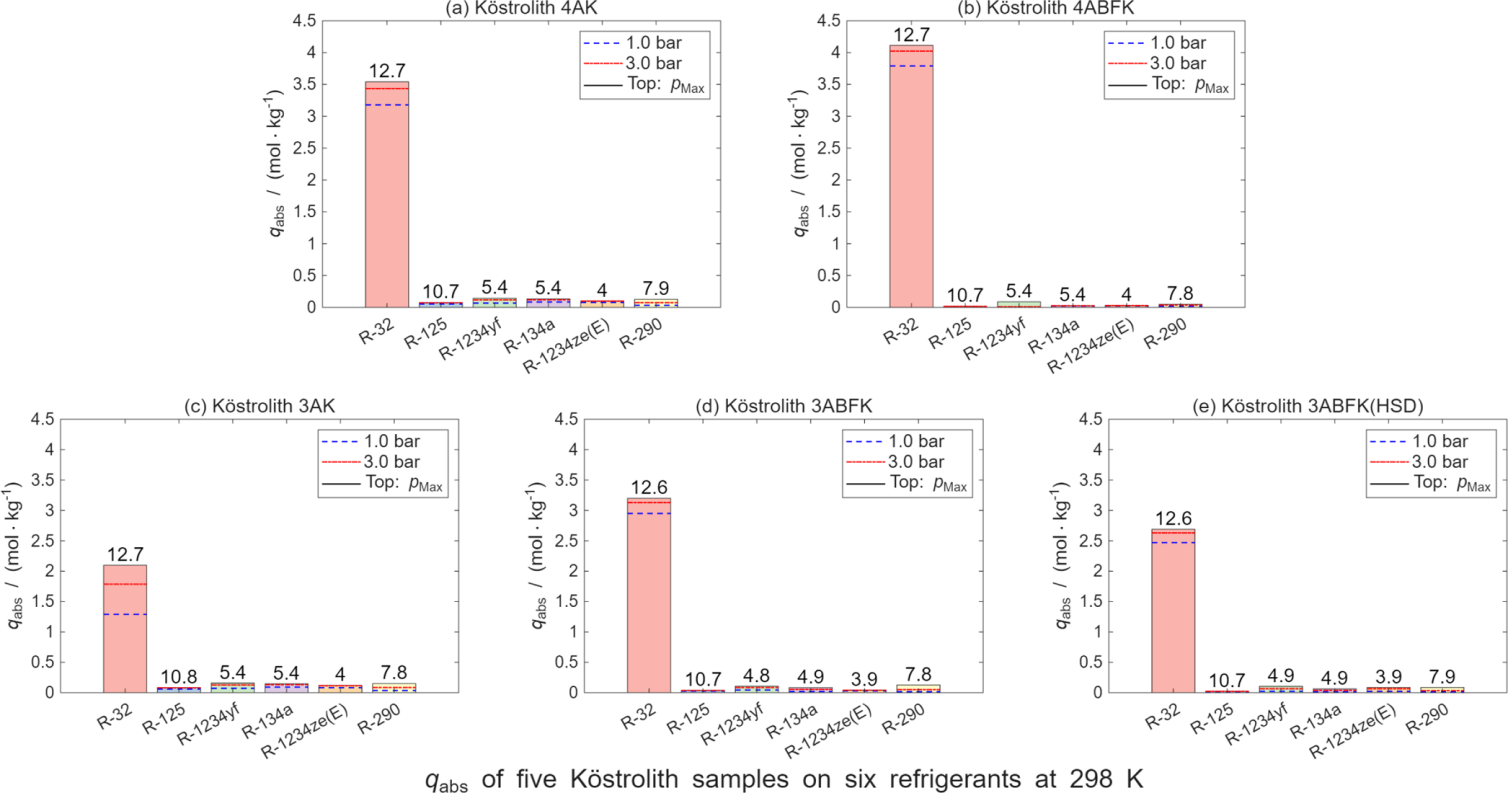
Absolute sorption capacities *q*_abs_ of six pure refrigerants on (a) Köstrolith 4AK, (b) Köstrolith 4ABFK, and (c) Köstrolith 3AK at *T* = 298 K; (d) Köstrolith 3ABFK and (e) Köstrolith 3ABFK­(HSD) at *T* = 303 K under different pressures *p* (1 bar, 3 bar, and *p*_Max_). Data obtained from Langmuir model fitting.

**Figure 10:**
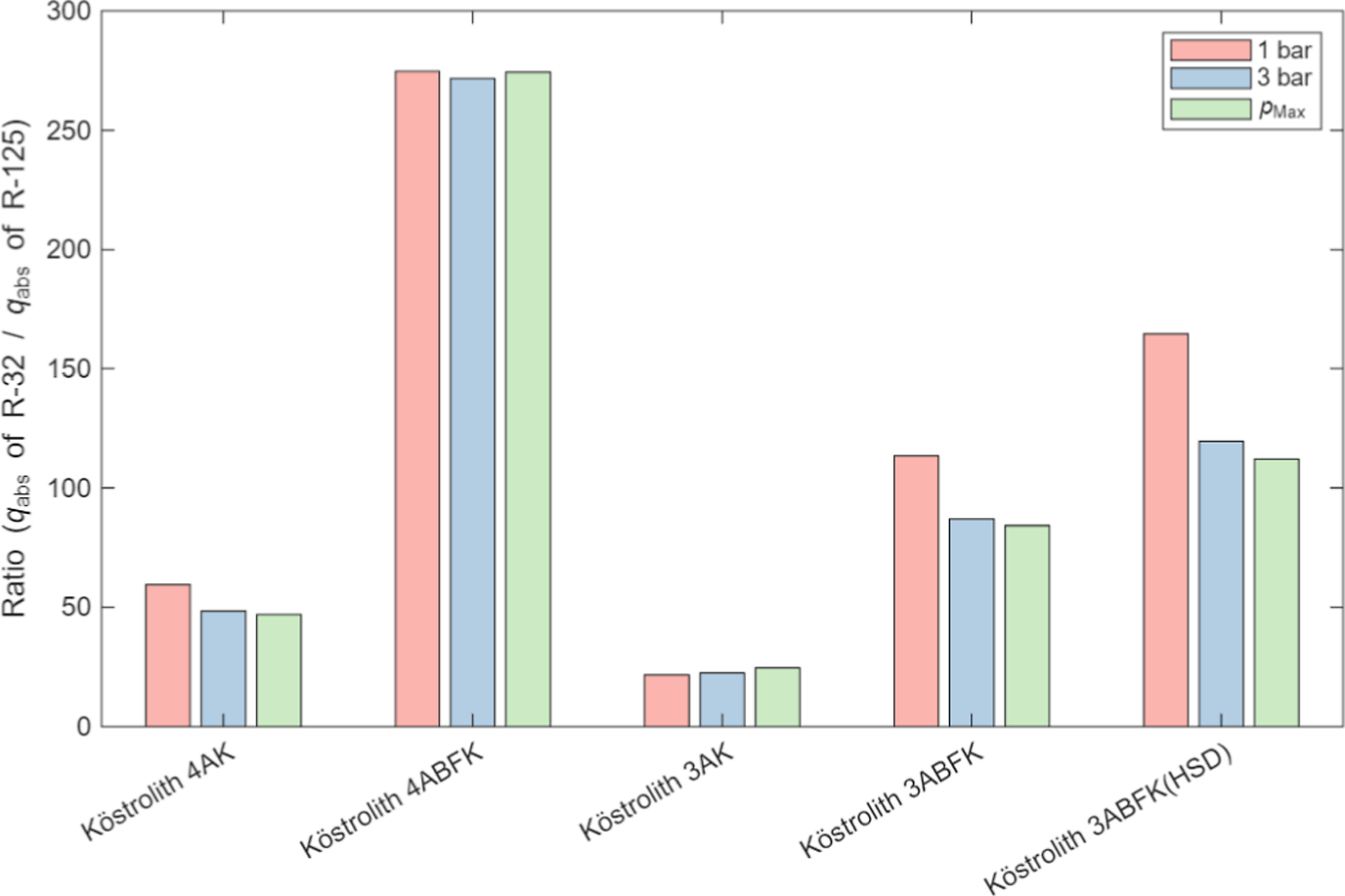
Adsorption ratios of R-32 and R-125 on the five zeolite materials (Köstrolith 4AK, Köstrolith 4ABFK, and Köstrolith 3AK at *T* = 298 K; Köstrolith 3ABFK and Köstrolith 3ABFK­(HSD) at *T* = 303 K) at different pressures *p* (1 bar, 3 bar, and *p*_Max_).

Among these zeolite adsorbents, Köstrolith 4AK and 4ABFK (zeolite 4A) exhibit exceptional selectivity and capacity for R-32 adsorption. This arises from a synergistic interplay of size-sieving and intense electrostatic interactions within its narrow 8-ring pore apertures. With a kinetic diameter of 3.9 Å, R-32 is the only refrigerant capable of activated diffusion into the 4A framework, while the remaining five larger molecules, e.g., R-290 (4.3 Å see Table 1), are effectively excluded. Furthermore, R-32’s high dipole moment (1.978 D see Table 1) and compact molecular volume enable close-range cation–dipole interactions with mobile Na^+^ ions in the SII sites, amplified by extreme pore confinement, resulting in high binding energies. In contrast, Köstrolith 3AK and 3ABFK variants, with smaller ∼ 3.0 Å pores, show lower uptakes for all refrigerants, underscoring the critical role of precise aperture matching in driving selective adsorption.

### 4.2 Other Samples

The absolute adsorption values *q*_abs_ of the three UiO-66 series samples, three MIL-100­(Fe) series samples, and one activated carbon sample for refrigerant gases at three pressures *p* (1 bar, 3 bar, and the highest measuring pressure) are shown in Figures 11–13, respectively. To reduce the size of the main manuscript, the adsorption isotherms and Langmuir model fitted tables of refrigerant gases on these materials are presented in Figures S17–S33, Tables S15–S31 in the Supporting Information, respectively.

**Figure 11:**
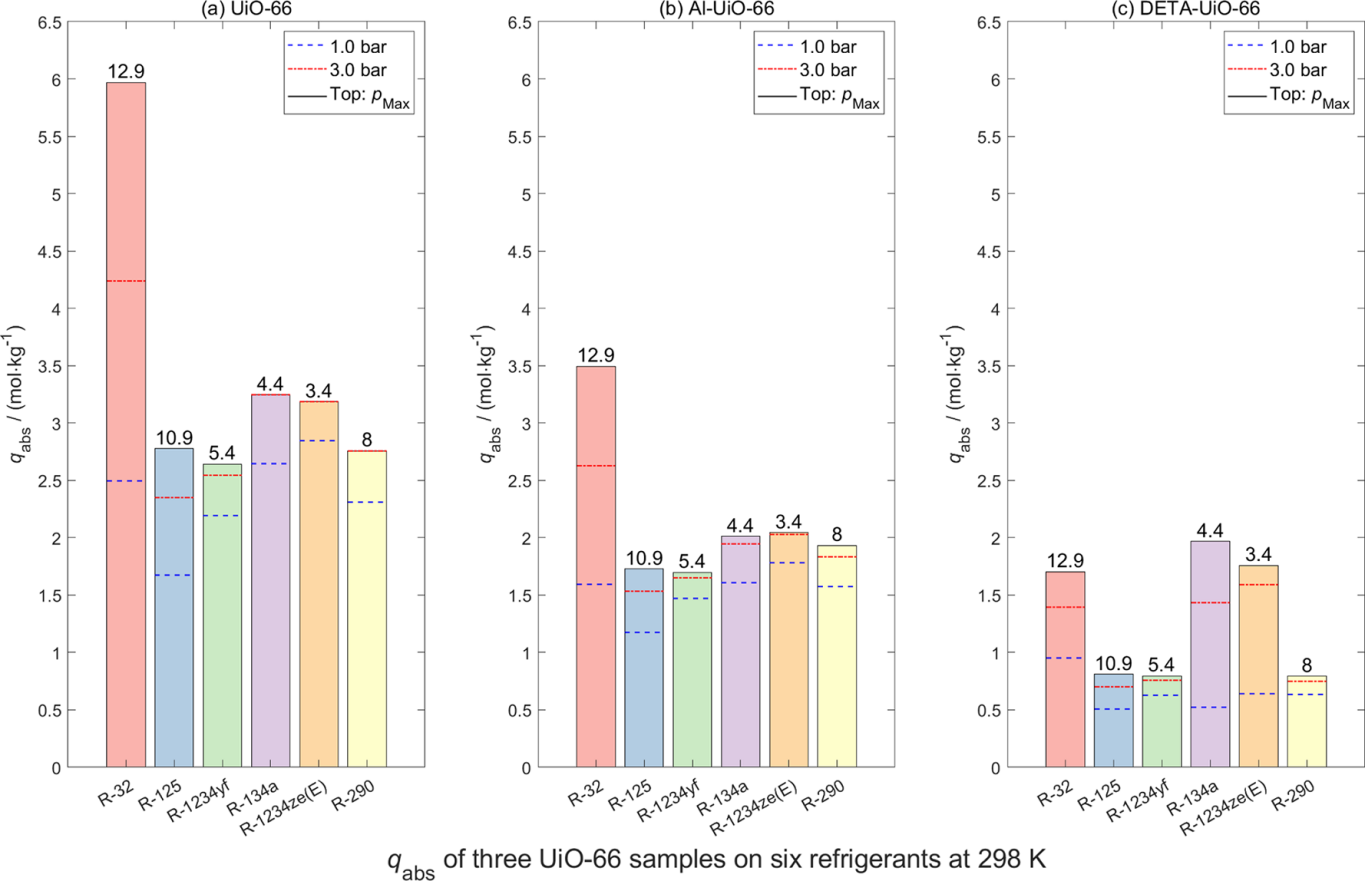
Absolute sorption capacities *q*_abs_ of six pure refrigerants on (a) UiO-66, (b) AI-UiO-66, and (c) DETA-UiO-66 at *T* = 298 K under different pressures *p* (1 bar, 3 bar, and *p*_Max_). Data obtained from Langmuir model fitting.

**12:**
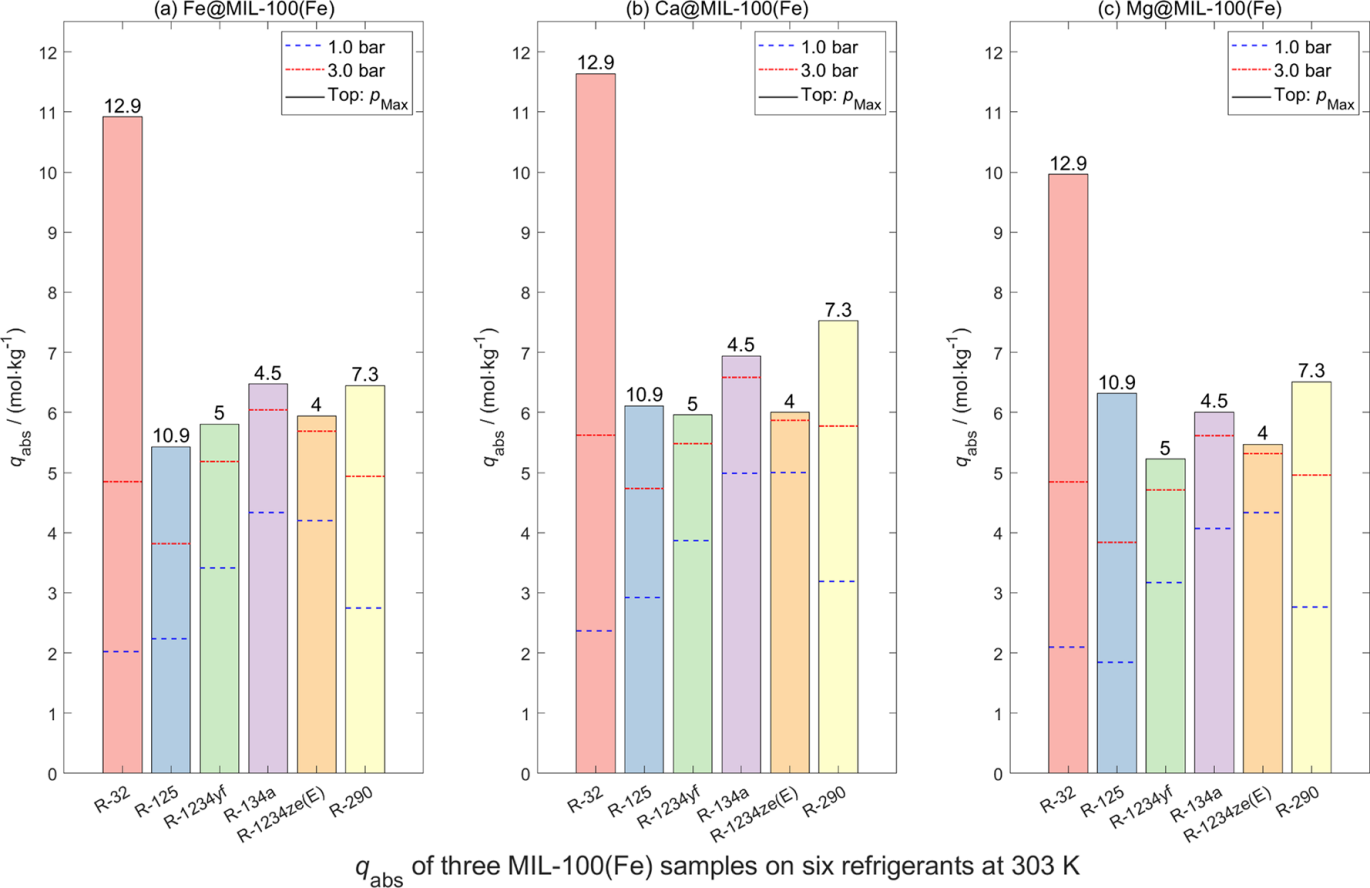
Absolute sorption capacities *q*_abs_ of six pure refrigerants on (a) Fe@MIL-100­(Fe), (b) Ca@MIL-100­(Fe), and (c) Mg@MIL-100­(Fe) at *T* = 303 K under different pressures *p* (1 bar, 3 bar, and *p*_Max_). Data obtained from Langmuir model fitting.

**Figure 13:**
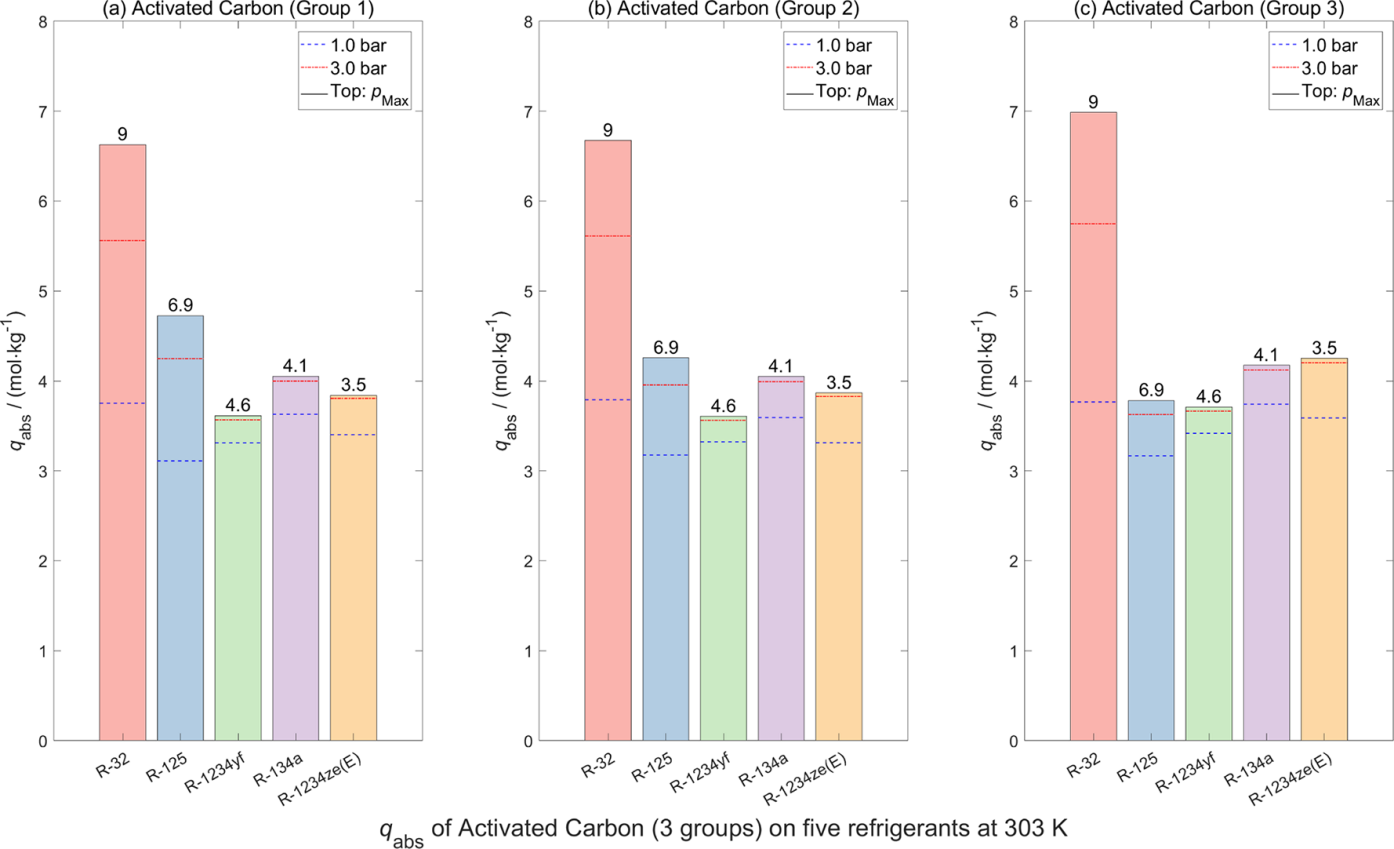
Absolute sorption capacities *q*_abs_ of five pure refrigerants on (a) activated carbon (group 1, *m* = 2.71254 g), (b) activated carbon (group 2, *m* = 2.21894 g), and (c) activated carbon (group 3, *m* = 1.91024 g) at *T* = 303 K under different pressures *p* (1 bar, 3 bar, and *p*_Max_). Data obtained from Langmuir model fitting.

As illustrated in these three figures, excluding DETA-UiO-66, the remaining six porous materials exhibit higher adsorption for R-32 than the other gases. At both 1 and 3 bar, the absolute adsorption capacities of R-32 and R-125 are comparable, as are those of R-1234yf and R-134a. This highlights the difficulty of separating R-32 from R-410A and R-1234yf from R-513A using these materials. In other words, these seven materials are not suitable for effective separation of these refrigerant mixtures. Nevertheless, the maximum *q*_abs_ for R-32 on MIL-100­(Fe) series samples approach or exceed 10 mol·kg^–1^, demonstrating their considerable potential for alternative applications such as gas storage. In addition, for the same activated carbon sample, adsorption measurements were conducted using different sample masses (2.71254, 2.21894, and 1.91024 g). As shown in Figure 13, slight variations in the adsorption data were observed, which can be attributed to the systematic errors in the experimental setup and the inherent uncertainties associated with volumetric measurements.

Compared to zeolite samples, the UiO-66 series, MIL-100­(Fe) series, and activated carbon exhibit higher R-32 uptake, largely due to their much larger BET surface areas (386–1860 m^2^/g see Table 2). However, their wider pore dimensions (5.5–30 Å) diminish both dispersive confinement and localized electrostatic field gradients, lowering interaction energies and resulting in largely nonselective adsorption across the refrigerant suite. While open metal sites in MIL-100 variants provide moderate enhancement via coordination effects, and amino-functionalized DETA-UiO-66 increases polarity, these materials cannot replicate the subnanometer confinement and cation-induced field strength characteristic of zeolite 4A. As a result, although MOFs and activated carbon excel in high-capacity physisorption of larger or less polar gases, they are substantially less effective than Köstrolith 4AK and 4ABFK for selective R-32 capture, underscoring the distinctive ability of small-pore zeolites to achieve molecular recognition through combined steric and electrostatic mechanisms.

## 5 Conclusion

A triple-port automatic high-pressure volumetric sorption analyzer (3HP-VSA, type: RuboSORP MPA-3, RUBOLAB) was set up for sorption measurements over the temperature range of 273.15 to 368.15 K at pressures up to 10 MPa. The main purpose of this apparatus is to rapidly screen porous materials for the separation of a specific gas mixture by measuring adsorption isotherms. To validate the new system, measurements with empty sample cells were first conducted to evaluate systematic errors in the system. Subsequently, sorption measurements of CO_2_ on Zeolite 4A, ZIF-8, and Zeolite 13X were performed, and the results were compared with literature data. Excellent agreement was achieved, demonstrating the high reliability of the new measurement system. A comprehensive uncertainty analysis was carried out to determine the uncertainty of the measurement results and to propose future improvements to the system.

Sorption measurements were then conducted on six pure refrigerants (R-32, R-125, R-1234yf, R-134a, R-1234ze­(E), and R-290) on 12 materials (Köstrolith 3AK, 3ABFK, 3ABFK­(HSD), 4AK, and 4ABFK; UiO-66, AI-UiO-66, and DETA-UiO-66; Fe@MIL-100­(Fe), Ca@MIL-100­(Fe), and Mg@MIL-100­(Fe); Alcarbon UC 50/4 × 8) at four temperatures (283, 298, 303, or 313 K) and pressures up to 0.1 MPa below the respective saturation pressures. All the zeolite samples investigated (Köstrolith 3AK, 3ABFK, 3ABFK­(HSD), 4AK, and 4ABFK) exhibited a relatively higher adsorption capacity for R-32 compared to the other five refrigerant gases, with Köstrolith 4ABFK demonstrating the highest selectivity. This characteristic allows for the potential use of any of these zeolite samples in the separation of R-32 from the R-410A blend (a mixture of R-32 and R-125, which is being phased out in the European Union), and in the separation of R-32 from mixtures comprising any of these six refrigerants. The other porous materials studied also showed relatively high adsorption capacities for R-32. However, their much lower selectivity over R-125 resulted in significantly lower performance compared to the zeolite samples. Furthermore, since these materials exhibited nearly identical adsorption capacities for R-125, R-290, R-1234yf, R-1234ze­(E), and R-134a, they did not produce any promising results for the separation of refrigerant blends among these five gases.

## Supplementary Material





## Data Availability

All data required to understand the present work are provided in the Supporting Information. Adsorption data are provided in the format of AIF (adsorption information format).

## Notes

All adsorption data are provided in the Adsorption Information File (AIF) format and as plain-text files including uncertainty information (ZIP)
Tables S1–S2: Gas specifications and samples information; Tables S3–S31: Langmuir fitting parameters for refrigerant adsorption on the investigated materials; Figures S1–S2: Measurements with empty measurement cell; Figures S3–S33: Refrigerant adsorption isotherms for the different samples (PDF)
