# Supplementary material for: A Triple-Port High-Pressure Volumetric Sorption Analyzer: Calibration, Measurements, and Uncertainty Analysis for Refrigerant Blend Separation Applications
Source: ACS Omega. 2026 Mar 6;11(11):17302–14. doi: 10.1021/acsomega.5c09911 (PMC13019387; doi:10.1021/acsomega.5c09911)
Supplement: Supplementary file 2 [file ao5c09911_si_002.pdf]

Supporting information

A triple-port high-pressure volumetric sorption analyzer: calibration, measurements, and uncertainty analysis for refrigerant blend separation applications

Gensheng Lin[1,2], Panpan Liu[3], Markus Richter[1,2], Xiaoxian Yang[1,*]

[1] *Chemnitz University of Technology, Applied Thermodynamics, 09107 Chemnitz, Germany*

[2] *Leibniz University Hannover, Institute of Thermodynamics, An der Universität 1, 30823 Garbsen, Germany*

[3] *Heze University, College of Pharmacy, Heze 274015, PR China*

* *Corresponding author. Email address: xiaoxian.yang@mb.tu-chemnitz.de*

1. Gas sample information

Table S1. Gas sample information.

Chemical name	Source	Purity/mole fraction	Purification method
Difluoromethane (R-32)	TEGA	0.995[a]	none
Tetrafluoropropene (R-1234yf)	TEGA	0.995[b]	none
Pentafluoroethane (R-125)	TEGA	0.995[c]	none
Propane (R-290)	TEGA	0.995[d]	none
Tetrafluoroethane (R-134a)	Westfalen	0.995[e]	none
trans-1,3,3,3-tetrafluoropropene (R-1234ze(E))	Westfalen	0.995[f]	none
Carbon dioxide (CO_2)	Air Liquid	0.99995[g]	none
Nitrogen (N_2)	Air Liquid	0.999999[h]	none
Argon (Ar)	Air Liquid	0.999999[i]	none
Helium (He)	Nippon Gases	0.999999[j]	none

[a] H_2O < 10 mg/kg (ppm), high boiling residue < 0.01 %-wt, acidity < 1 mg/kg, non-condensable gases < 1.5 %-vol.

[b] H_2O < 20 mg/kg (ppm), high boiling residue < 0.01 %-wt, acidity < 1 mg/kg, non-condensable gases < 1.5 %-vol.

[c] H_2O < 10 mg/kg (ppm), residue < 0.01 %-wt, acidity < 1 mg/kg, non-condensable gases < 1.5 %-vol.

[d] H_2O < 10 mg/kg (ppm), residue < 0.01 %-wt, acidity < 1 mg/kg, non-condensable gases < 1.5 %-vol, n-hexane < 1 mg/kg (ppm), 1,3-butadiene < 1 mg/kg (ppm).

[e] No information available from the provider.

[f] No information available from the provider.

[g] H_2O < 2 ppmv, O_2 < 1 ppmv, KW < 0.1 ppmv, N_2 < 2 ppmv, CO < 0.5 ppmv, NO_x 0.1 ppmv.

[h] Impurities: H_2O ⩽ 0.5 mol ppm, O_2 ⩽ 0.1 mol ppm, C_nH_m ⩽ 0.1 mol ppm, CO ⩽ 0.1 mol ppm, CO_2 ⩽ 0.1 mol ppm, H_2 ⩽ 0.1 mol ppm, Halothane ⩽ 0.001 mol ppm.

[i] Impurities (stated by supplier): H_2O ⩽ 0.5 mol ppm, O_2 ⩽ 0.1 mol ppm, CO ⩽ 0.1 mol ppm, CO_2 ⩽ 0.1 mol ppm, C_nH_m ⩽ 0.1 mol ppm, H_2 ⩽ 0.1 mol ppm, KW ⩽ 1 mol ppm.

[j] Impurities (stated by supplier): H_2O ≤ 0.5 mol ppm, O_2 ≤ 0.1 mol ppm, CO ≤ 0.1 mol ppm, CO_2 ≤ 0.1 mol ppm, C_nH_m ≤ 0.1 mol ppm, H_2 ≤ 0.1 mol ppm, Halothane ≤ 1 mol ppm.

Table S2. Activation induced mass loss $\varphi_{activate}$ of Zeolite 4A, Zeolite 4ABFK, ZIF-8, and Zeolite 13X.

Sample name	Before testing: *m* / g	After testing: *m* / g	Weight loss(%)
Zeolite 4A	4.114387	4.076120	0.93
Zeolite 4A	2.560666	2.526514	1.33
Zeolite 4ABFK	3.954467	3.952081	0.06
ZIF-8	1.442619	1.385252	3.98
Zeolite 13X	4.735761	4.641045	2.00

2. Measurements with empty measurement cell

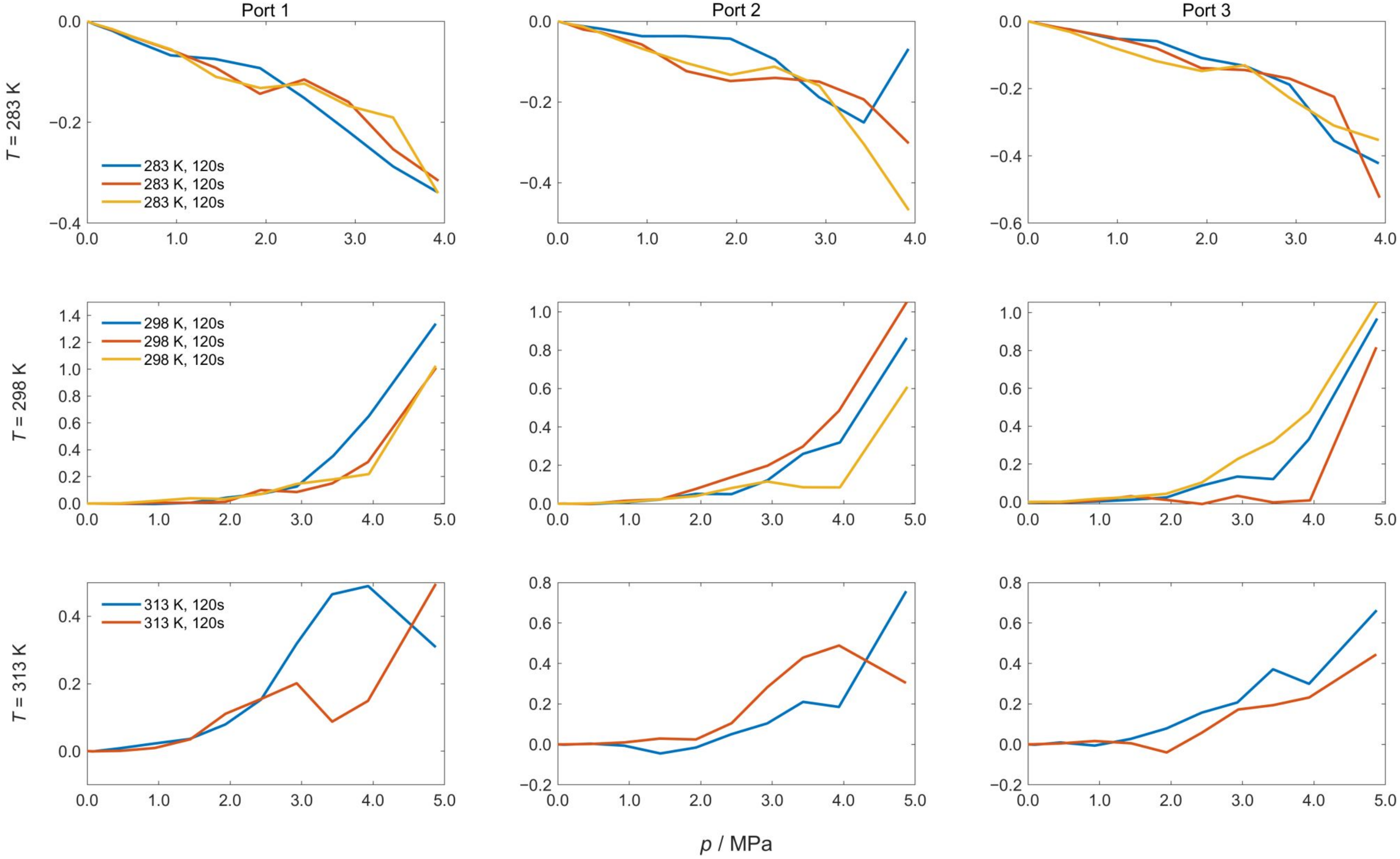

Figure S1: Isothermal adsorption curves of CO_2 obtained from empty measurements, assuming a nominal sample mass of 6 g for reference purposes.

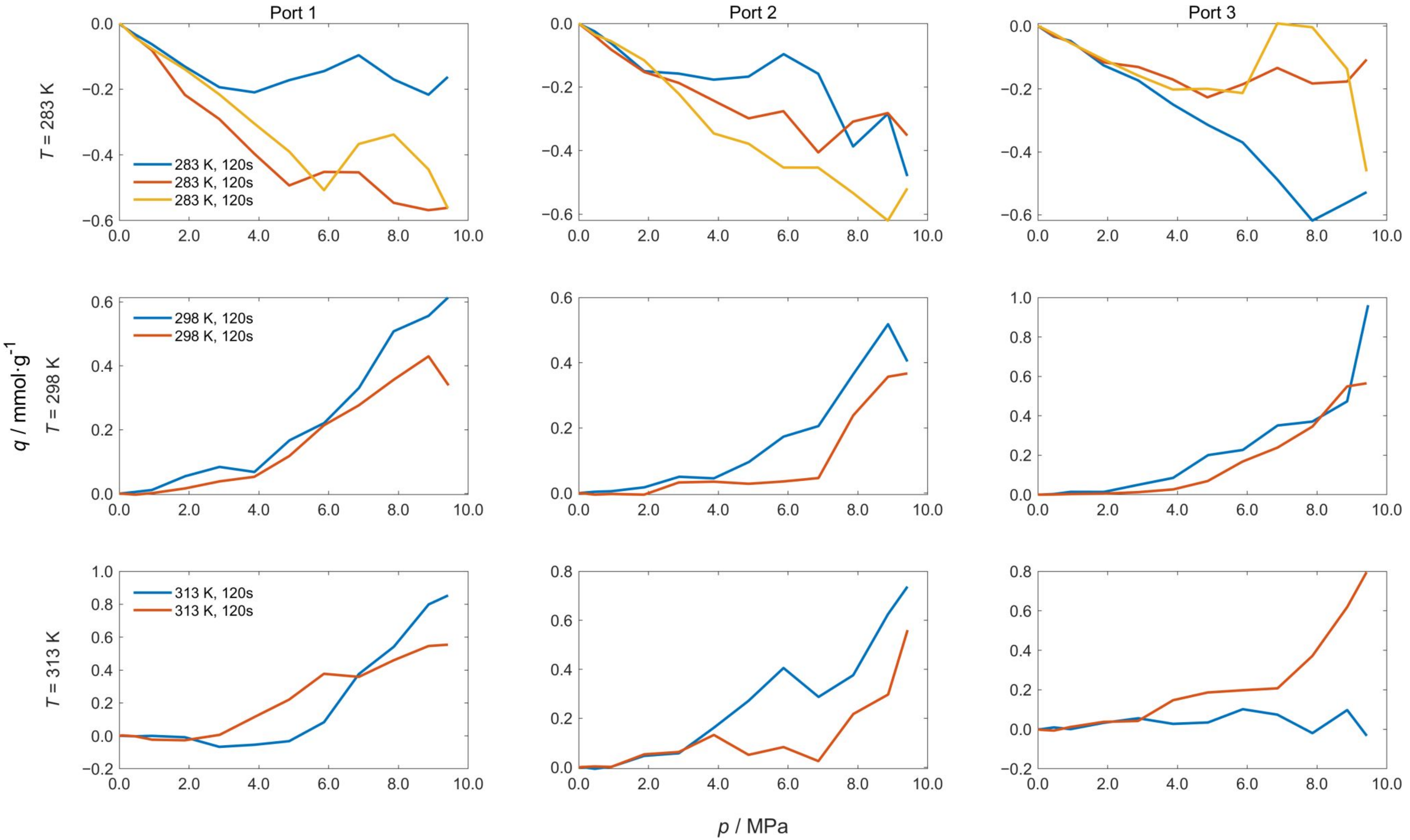

Figure S2: Isothermal adsorption curves of Ar obtained from empty measurements, assuming a nominal sample mass of 6 g for reference purposes.

3. Characterization of porous materials

3.1 Adsorption data for zeolite samples

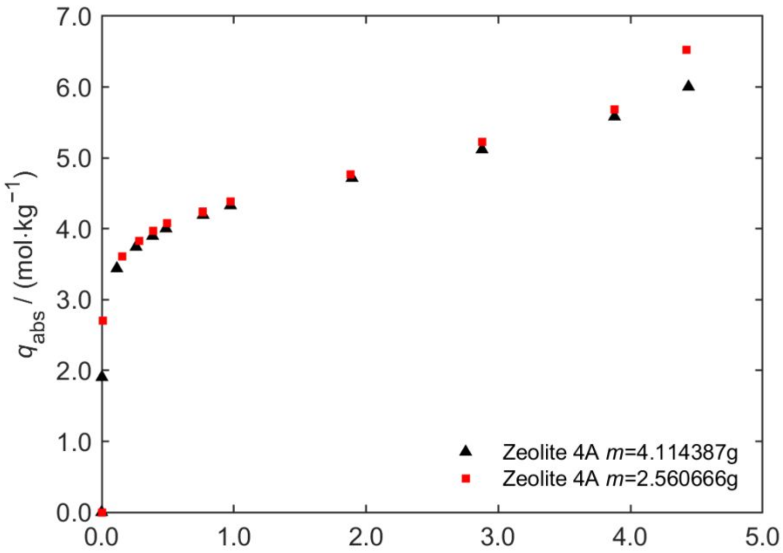

Figure S3: Adsorption isotherms of CO_2 at 283 K on different weights of Zeolite 4A samples. ▲, m = 4.114387 g; ■, m = 2.560666 g.

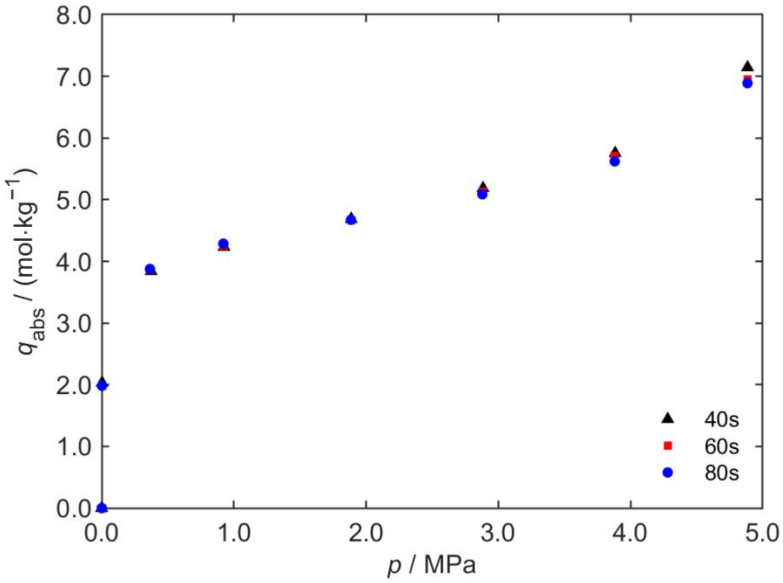

Figure S4: Adsorption isotherms of CO_2 on Zeolite 4A at 298 K with different equilibrium conditions: less than 1.0 kPa change in ▲, t = 40 s; ■, t = 60 s; and ●, t = 80 s.

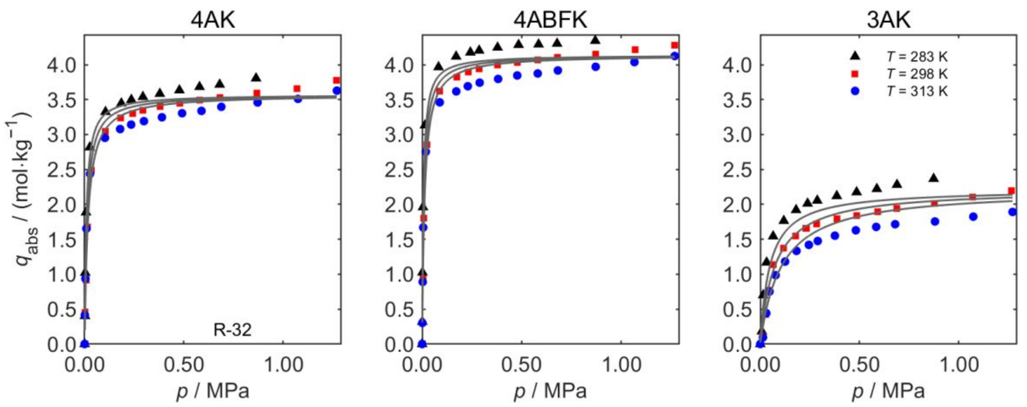

Figure S5: Absolute sorption capacity q_{abs} of the pure refrigerant R-32 on Köstrolith 4AK, 4ABFK, and 3AK at different pressures p. ▲, T = 283 K; ■, T = 298 K; ●, T = 313 K. Pressure range: 0 ~ 1.27 MPa. The solid curves are the fits of the Langmuir model to the experimental data.

Table S3. Langmuir parameters of R-32 on three materials (Köstrolith 4AK, 4ABFK, and 3AK) and their standard statistical uncertainties, u, together with the Root Mean Square Deviation (RMSD) of the fit.

parameters	Köstrolith 4AK		Köstrolith 4ABFK		Köstrolith 3AK	
	value	u	value	u	value	u
q_m (mol·kg^{-1})	3.574	0.143	4.147	0.157	2.220	0.124
10^5 K (MPa^{-1})	8432	29819	7901	2758	3141	9360
ΔH (KJ·mol^{-1})	-16.997	8.737	-23.581	8.615	-20.782	7.442
RMSD (mol·kg^{-1})	0.173		0.191		0.135	

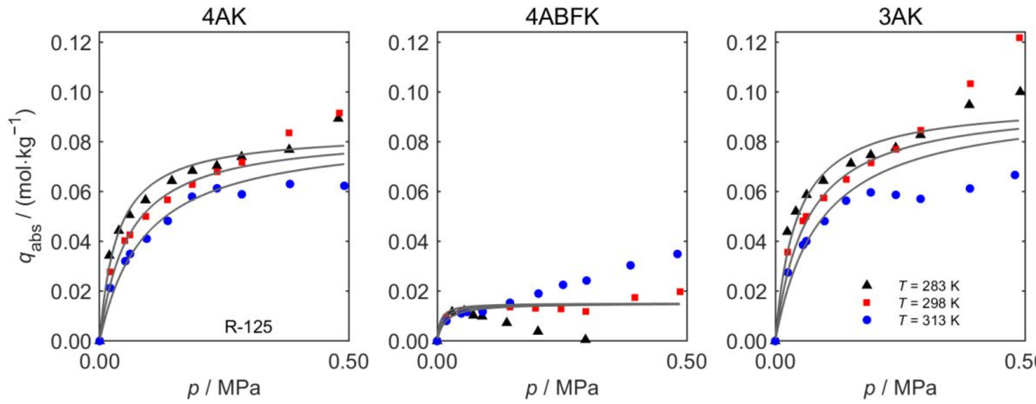

Figure S6: Absolute sorption capacity q_{abs} of the pure refrigerant R-125 on Köstrolith 4AK, 4ABFK, and 3AK at different pressures p. ▲, T = 283 K; ■, T = 298 K; ●, T = 313 K. Pressure range: 0 ~ 0.50 MPa. The solid curves are the fits of the Langmuir model to the experimental data.

Table S4. Langmuir parameters of R-125 on three materials (Köstrolith 4AK, 4ABFK, and 3AK) and their standard statistical uncertainties, u, together with the Root Mean Square Deviation (RMSD) of the fit.

parameters	Köstrolith 4AK	Köstrolith 4ABFK	Köstrolith 3AK

	value	u	value	u	value	u
q_m (mol·kg^{-1})	0.0845	0.0066	0.0158	0.0076	0.0962	0.0131
10^5 K (MPa^{-1})	1883	5417	11014	408820	112	555
ΔH (KJ·mol^{-1})	-16.853	7.171	-15.942	93.700	-23.730	12.434
RMSD (mol·kg^{-1})	0.005		0.007		0.009	

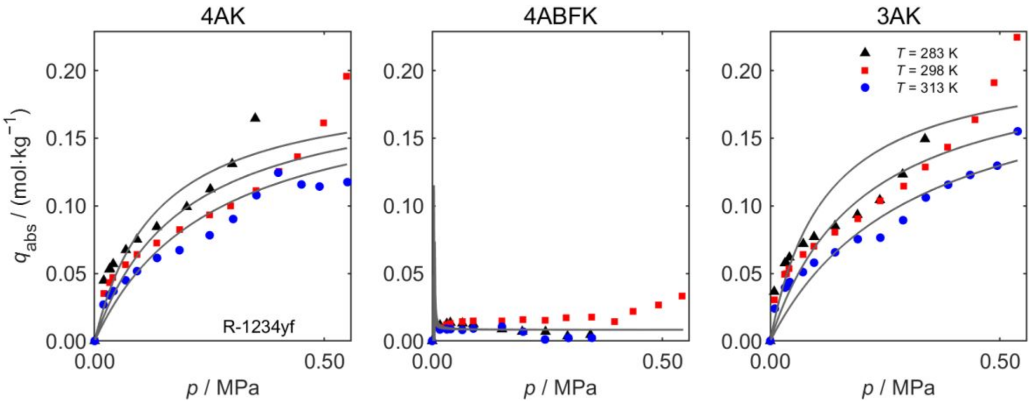

Figure S7: Absolute sorption capacity q_{abs} of the pure refrigerant R-1234yf on Köstrolith 4AK, 4ABFK, and 3AK at different pressures p. ▲, T = 283 K; ■, T = 298 K; ●, T = 313 K. Pressure range: 0 ~ 0.55 MPa. The solid curves are the fits of the Langmuir model to the experimental data.

Table S5. Langmuir parameters of R-1234yf on three materials (Köstrolith 4AK, 4ABFK, and 3AK) and their standard statistical uncertainties, u, together with the Root Mean Square Deviation (RMSD) of the fit.

parameters	Köstrolith 4AK		Köstrolith 4ABFK		Köstrolith 3AK	
	value	u	value	u	value	u
q_m (mol·kg^{-1})	0.1918	0.0299	0.0082	0.0080	0.2120	0.0432
10^5 K (MPa^{-1})	879	2542	-14390	130650	31	109
ΔH (KJ·mol^{-1})	-15.866	7.137	-18.676	233.780	-24.025	8.8647
RMSD (mol·kg^{-1})	0.014		0.010		0.018	

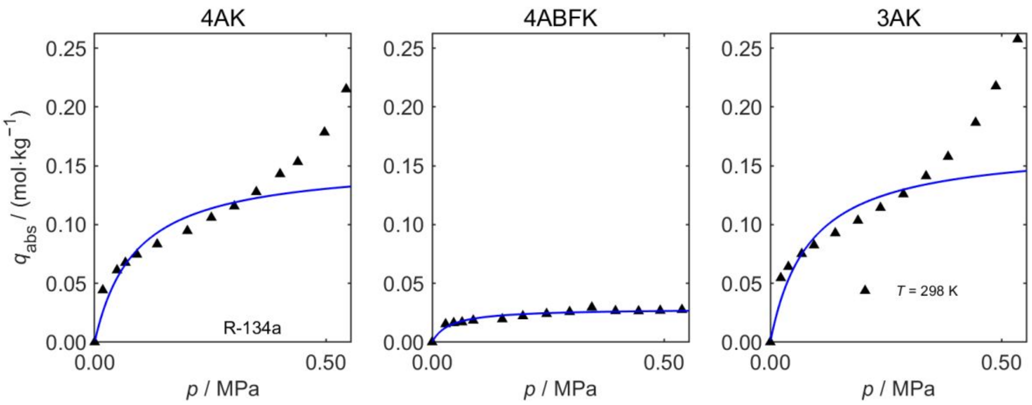

Figure S8: Absolute sorption capacity q_{abs} of the pure refrigerant R-134a on Köstrolith 4AK, 4ABFK, and 3AK at different pressures p. ▲, T = 298 K. Pressure range: 0 ~ 0.55 MPa. The solid curves are the fits of the Langmuir model to the experimental data.

Table S6. Langmuir parameters of R-134a on three materials (Köstrolith 4AK, 4ABFK, and 3AK) and their standard statistical uncertainties, u, together with the Root Mean Square Deviation (RMSD) of the fit.

parameters	Köstrolith 4AK		Köstrolith 4ABFK		Köstrolith 3AK	
	value	u	value	u	value	u
q_m (mol·kg^{-1})	0.1533	0.0279	0.0285	0.0035	0.1678	0.0324
10^5 K (MPa^{-1})	1138970	640510	2617910	1405580	1184680	705410
RMSD (mol·kg^{-1})	0.009		0.002		0.011	

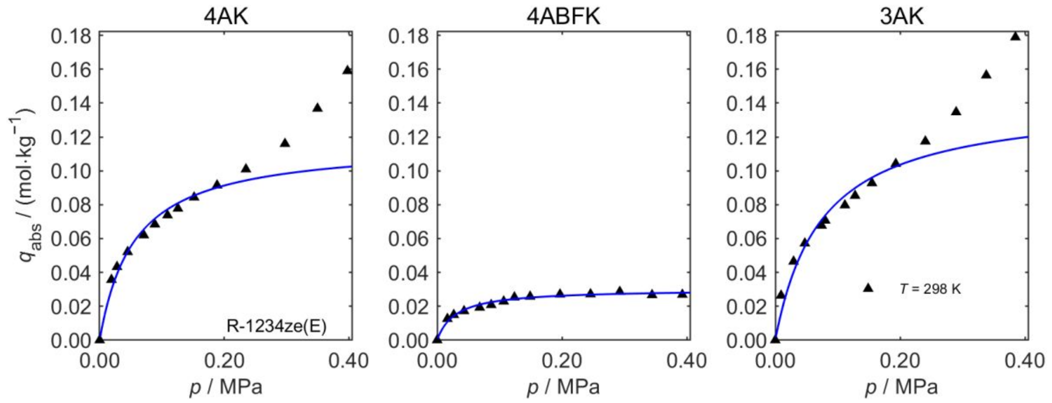

Figure S9: Absolute sorption capacity q_{abs} of the pure refrigerant R-1234ze(E) on Köstrolith 4AK, 4ABFK, and 3AK at different pressures p. ▲, T = 298 K. Pressure range: 0 ~ 0.40 MPa. The solid curves are the fits of the Langmuir model to the experimental data.

Table S7. Langmuir parameters of R-1234ze(E) on three materials (Köstrolith 4AK, 4ABFK, and 3AK) and their standard statistical uncertainties, u, together with the Root Mean Square Deviation (RMSD) of the fit.

parameters	Köstrolith 4AK		Köstrolith 4ABFK		Köstrolith 3AK	
	value	u	value	u	value	u
q_m (mol·kg^{-1})	0.1166	0.0113	0.0285	0.0035	0.1678	0.0324
10^5 K (MPa^{-1})	1804770	507220	2617910	1405580	1184680	705410
RMSD (mol·kg^{-1})	0.009		0.002		0.011	

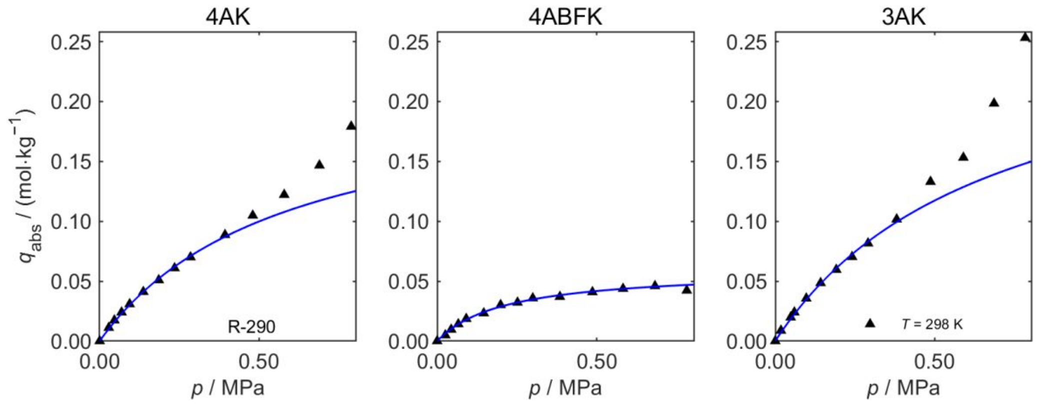

Figure S10: Absolute sorption capacity q_{abs} of the pure refrigerant R-290 on Köstrolith 4AK, 4ABFK, and 3AK at different pressures p. ▲, T = 298 K. Pressure range: 0 ~ 0.80 MPa. The

solid curves are the fits of the Langmuir model to the experimental data.

Table S8. Langmuir parameters of R-290 on three materials (Köstrolith 4AK, 4ABFK, and 3AK) and their standard statistical uncertainties, *u*, together with the Root Mean Square Deviation (RMSD) of the fit.

parameters	Köstrolith 4AK		Köstrolith 4ABFK		Köstrolith 3AK	
	value	*u*	value	*u*	value	*u*
q_m (mol·kg^{-1})	0.2161	0.0292	0.0597	0.0070	0.2763	0.0532
10^5 *K* (MPa^{-1})	171740	33300	467040	111070	1147580	39240
RMSD (mol·kg^{-1})	0.001		0.001		0.001	

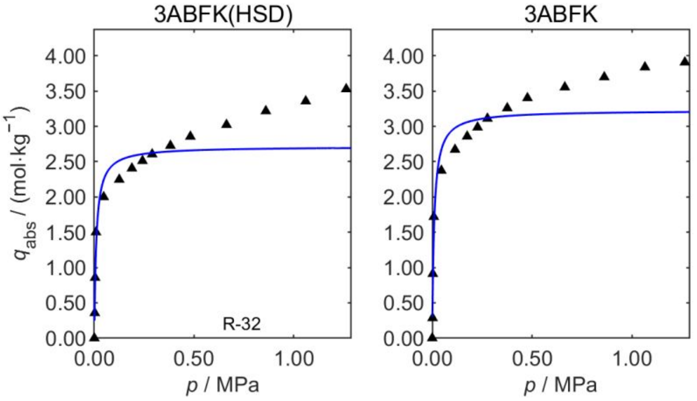

Figure S11: Absolute sorption capacity q_{abs} of the pure refrigerant R-32 on Köstrolith 3ABFK(HSD) and 3ABFK at different pressures *p*. ▲, *T* = 303 K. Pressure range: 0 ~ 1.28 MPa. The solid curves are the fits of the Langmuir model to the experimental data.

Table S9. Langmuir parameters of R-32 on two materials (Köstrolith 3ABFK(HSD) and 3ABFK) and their standard statistical uncertainties, *u*, together with the Root Mean Square Deviation (RMSD) of the fit.

parameters	Köstrolith 3ABFK(HSD)		Köstrolith 3ABFK	
	value	*u*	value	*u*
q_m (mol·kg^{-1})	2.7137	0.1852	3.2274	0.2169
10^5 *K* (MPa^{-1})	10107790	4755790	10449530	5095540
RMSD (mol·kg^{-1})	0.183		0.212	

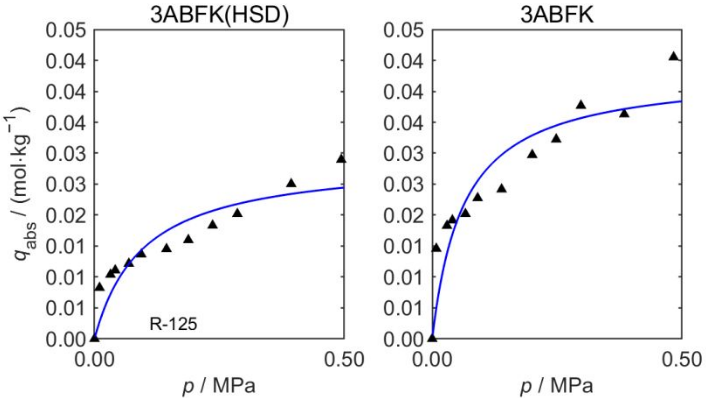

Figure S12: Absolute sorption capacity q_{abs} of the pure refrigerant R-125 on Köstrolith 3ABFK(HSD) and 3ABFK at different pressures p. ▲, T = 303 K. Pressure range: 0 ~ 0.50 MPa. The solid curves are the fits of the Langmuir model to the experimental data.

Table S10. Langmuir parameters of R-125 on two materials (Köstrolith 3ABFK(HSD) and 3ABFK) and their standard statistical uncertainties, u, together with the Root Mean Square Deviation (RMSD) of the fit.

parameters	Köstrolith 3ABFK(HSD)		Köstrolith 3ABFK	
	value	u	value	u
q_m (mol·kg^{-1})	0.0292	0.0079	0.0432	0.0092
10^5 K (MPa^{-1})	1028950	815580	1587880	1190440
RMSD (mol·kg^{-1})	0.003		0.004	

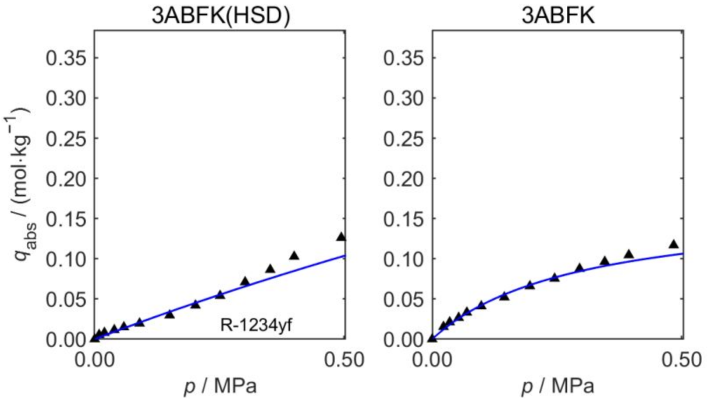

Figure S13: Absolute sorption capacity q_{abs} of the pure refrigerant R-1234yf on Köstrolith 3ABFK(HSD) and 3ABFK at different pressures p. ▲, T = 303 K. Pressure range: 0 ~ 0.50 MPa. The solid curves are the fits of the Langmuir model to the experimental data.

Table S11. Langmuir parameters of R-1234yf on two materials (Köstrolith 3ABFK(HSD) and 3ABFK) and their standard statistical uncertainties, u, together with the Root Mean Square Deviation (RMSD) of the fit.

parameters	Köstrolith 3ABFK(HSD)		Köstrolith 3ABFK	
	value	u	value	u
q_m (mol·kg^{-1})	0.8655	2.9677	0.1687	0.0287
10^5 K (MPa^{-1})	27180	99230	338670	94660
RMSD (mol·kg^{-1})	0.003		0.002	

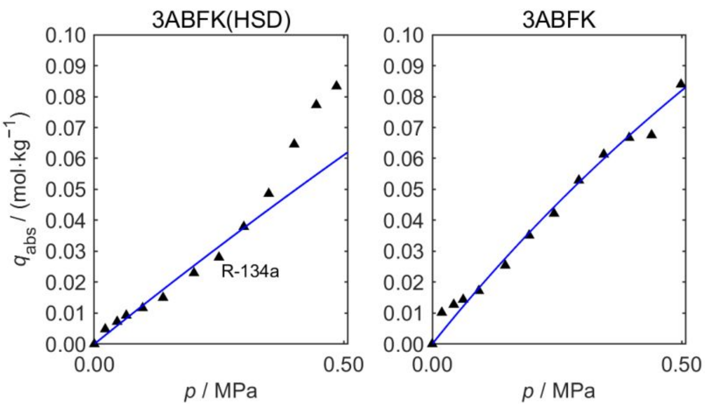

Figure S14: Absolute sorption capacity q_{abs} of the pure refrigerant R-134a on Köstrolith 3ABFK(HSD) and 3ABFK at different pressures p. ▲, T = 303 K. Pressure range: 0 ~ 0.50 MPa. The solid curves are the fits of the Langmuir model to the experimental data.

Table S12. Langmuir parameters of R-134a on two materials (Köstrolith 3ABFK(HSD) and 3ABFK) and their standard statistical uncertainties, u, together with the Root Mean Square Deviation (RMSD) of the fit.

parameters	Köstrolith 3ABFK(HSD)		Köstrolith 3ABFK	
	value	u	value	u
q_m (mol·kg^{-1})	0.9998	8.0678	0.4341	0.8446
10^5 K (MPa^{-1})	13020	108840	46560	101710
RMSD (mol·kg^{-1})	0.002		0.003	

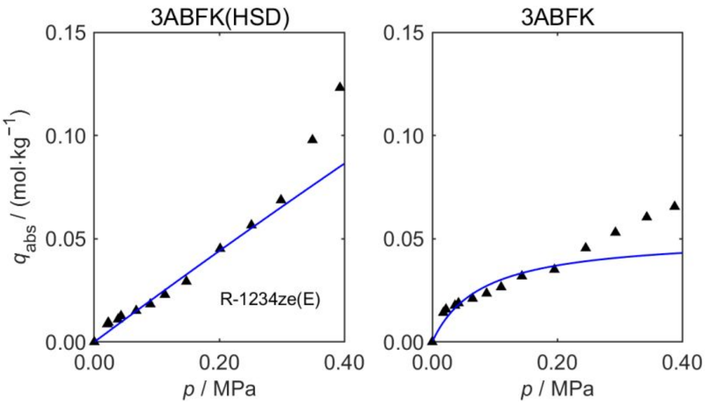

Figure S15: Absolute sorption capacity q_{abs} of the pure refrigerant R-1234ze(E) on Köstrolith 3ABFK(HSD) and 3ABFK at different pressures p. ▲, T = 303 K. Pressure range: 0 ~ 0.40 MPa. The solid curves are the fits of the Langmuir model to the experimental data.

Table S13. Langmuir parameters of R-1234ze(E) on two materials (Köstrolith 3ABFK(HSD) and 3ABFK) and their standard statistical uncertainties, u, together with the Root Mean Square Deviation (RMSD) of the fit.

parameters	Köstrolith 3ABFK(HSD)		Köstrolith 3ABFK	
	value	u	value	u
q_m (mol·kg^{-1})	2.0961	21.6899	0.0515	0.0136
10^5 K (MPa^{-1})	10740	113450	1279490	809290

RMSD ($mol \cdot kg^{-1}$)	0.002	0.003

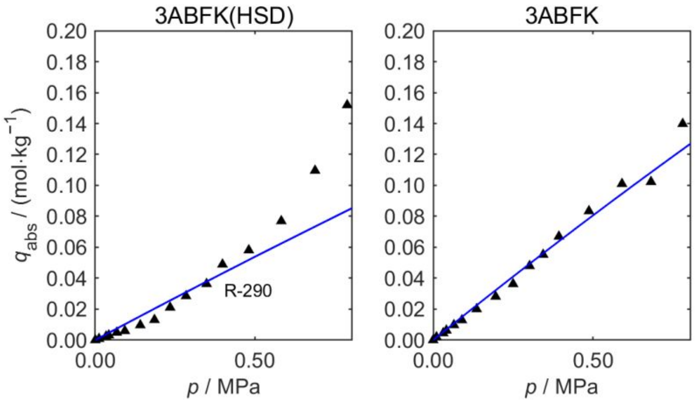

Figure S16: Absolute sorption capacity q_{abs} of the pure refrigerant R-290 on Köstrolith 3ABFK(HSD) and 3ABFK at different pressures p. ▲, T = 303 K. Pressure range: 0 ~ 0.80 MPa. The solid curves are the fits of the Langmuir model to the experimental data.

Table S14. Langmuir parameters of R-290 on two materials (Köstrolith 3ABFK(HSD) and 3ABFK) and their standard statistical uncertainties, u, together with the Root Mean Square Deviation (RMSD) of the fit.

parameters	Köstrolith 3ABFK(HSD)		Köstrolith 3ABFK	
	value	u	value	u
q_m ($mol \cdot kg^{-1}$)	2.1043	34.7224	2.2517	11.1706
10^5 K (MPa^{-1})	5250	88220	7430	37840
RMSD ($mol \cdot kg^{-1}$)	0.004		0.002	

3.2 Adsorption data for UiO-66 samples

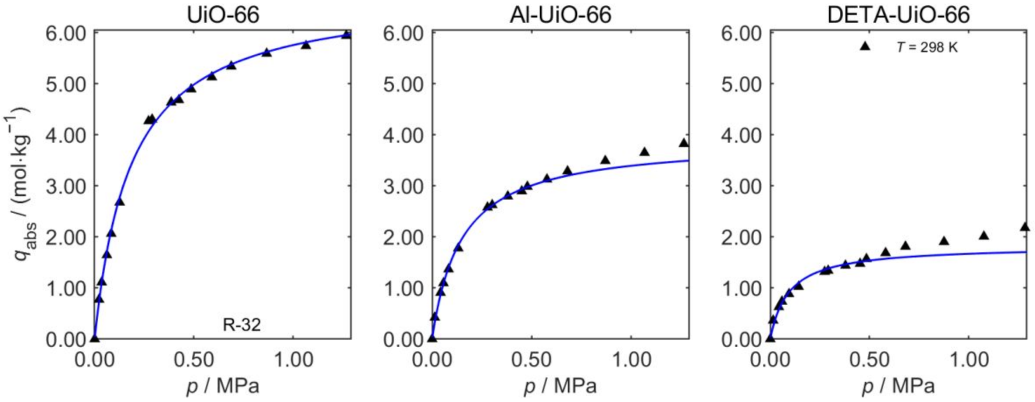

Figure S17: Absolute sorption capacity q_{abs} of the pure refrigerant R-32 on UiO-66, Al-UiO-66, and DETA-UiO-66 at different pressures p. ▲, T = 298 K. Pressure range: 0 ~ 1.30 MPa. The solid curves are the fits of the Langmuir model to the experimental data.

Table S15. Langmuir parameters of R-32 on three materials (UiO-66, Al-UiO-66, and DETA-UiO-66) and their standard statistical uncertainties, u, together with the Root Mean Square Deviation (RMSD) of the fit.

parameters	UiO-66		Al-UiO-66		DETA-UiO-66	
	value	u	value	u	value	u
q_m (mol·kg^{-1})	6.8163	0.3280	3.8938	0.1188	1.8265	0.1318
10^5 K (MPa^{-1})	544770	69640	679750	61000	1068000	271890
RMSD (mol·kg^{-1})	0.085		0.037		0.058	

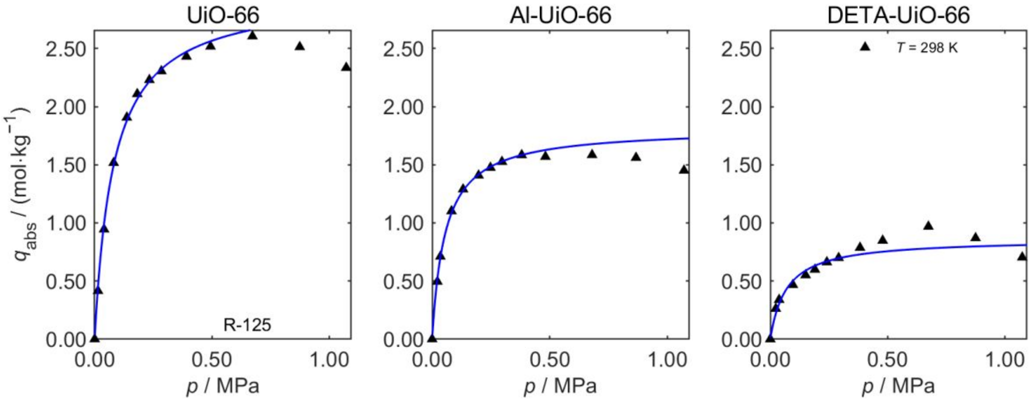

Figure S18: Absolute sorption capacity q_{abs} of the pure refrigerant R-125 on UiO-66, Al-UiO-66, and DETA-UiO-66 at different pressures p. ▲, T = 298 K. Pressure range: 0 ~ 1.10 MPa. The solid curves are the fits of the Langmuir model to the experimental data.

Table S16. Langmuir parameters of R-125 on three materials (UiO-66, Al-UiO-66, and DETA-UiO-66) and their standard statistical uncertainties, u, together with the Root Mean Square Deviation (RMSD) of the fit.

parameters	UiO-66		Al-UiO-66		DETA-UiO-66	
	value	u	value	u	value	u
q_m (mol·kg^{-1})	2.9797	0.1071	1.8144	0.0330	0.8606	0.1024
10^5 K (MPa^{-1})	1244950	141070	1801620	120900	1446190	585890
RMSD (mol·kg^{-1})	0.029		0.012		0.032	

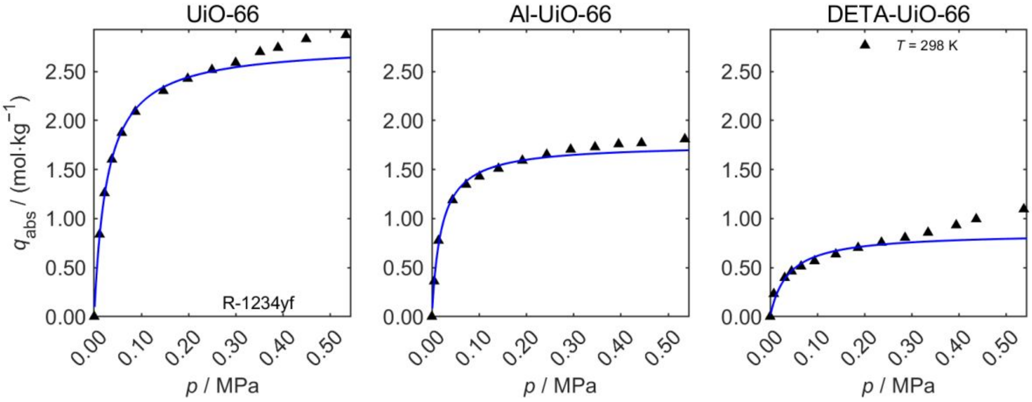

Figure S19: Absolute sorption capacity q_{abs} of the pure refrigerant R-1234yf on UiO-66, Al-UiO-66, and DETA-UiO-66 at different pressures p. ▲, T = 298 K. Pressure range: 0 ~ 0.54 MPa. The solid curves are the fits of the Langmuir model to the experimental data.

Table S17. Langmuir parameters of R-1234yf on three materials (UiO-66, Al-UiO-66, and

DETA-UiO-66) and their standard statistical uncertainties, *u*, together with the Root Mean Square Deviation (RMSD) of the fit.

parameters	UiO-66		Al-UiO-66		DETA-UiO-66	
	value	*u*	value	*u*	value	*u*
q_m (mol·kg^{-1})	2.7738	0.0419	1.7583	0.0507	0.8527	0.0836
10^5 K (MPa^{-1})	3668180	226970	5026120	737330	2641620	959460
RMSD (mol·kg^{-1})	0.022		0.030		0.035	

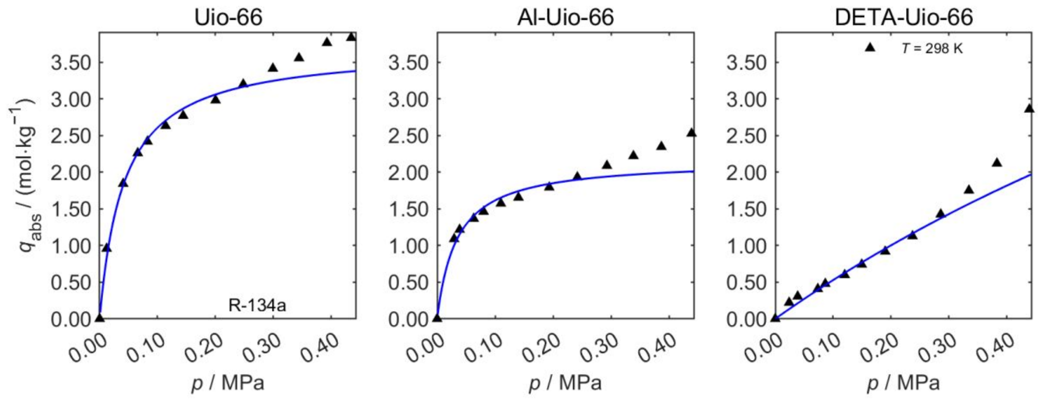

Figure S20: Absolute sorption capacity q_{abs} of the pure refrigerant R-134a on UiO-66, Al-UiO-66, and DETA-UiO-66 at different pressures *p*. ▲, T = 298 K. Pressure range: 0 ~ 0.44 MPa. The solid curves are the fits of the Langmuir model to the experimental data.

Table S18. Langmuir parameters of R-134a on three materials (UiO-66, Al-UiO-66, and DETA-UiO-66) and their standard statistical uncertainties, *u*, together with the Root Mean Square Deviation (RMSD) of the fit.

parameters	UiO-66		Al-UiO-66		DETA-UiO-66	
	value	*u*	value	*u*	value	*u*
q_m (mol·kg^{-1})	3.7025	0.2066	2.1665	0.1688	9.7338	13.7820
10^5 K (MPa^{-1})	2383810	498090	2934950	907170	57390	91140
RMSD (mol·kg^{-1})	0.079		0.071		0.047	

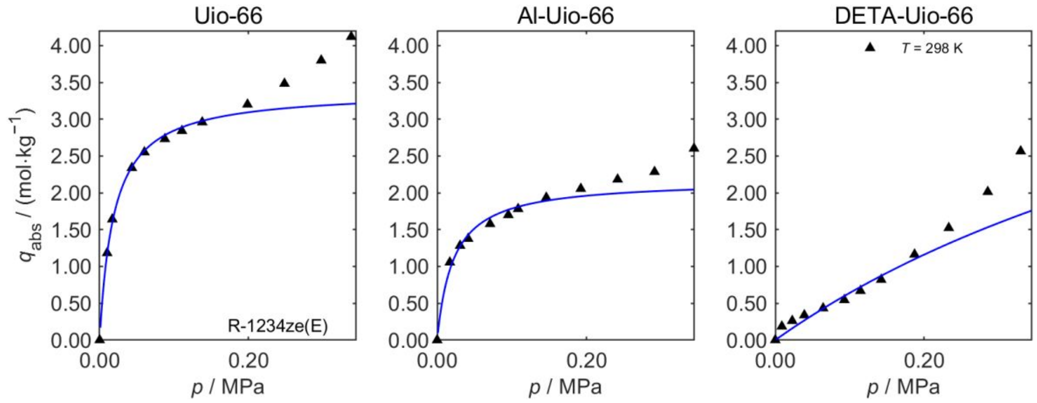

Figure S21: Absolute sorption capacity q_{abs} of the pure refrigerant R-1234ze(E) on UiO-66, Al-UiO-66, and DETA-UiO-66 at different pressures *p*. ▲, T = 298 K. Pressure range: 0 ~ 0.34 MPa. The solid curves are the fits of the Langmuir model to the experimental data.

Table S19. Langmuir parameters of R-1234ze(E) on three materials (UiO-66, Al-UiO-66, and DETA-UiO-66) and their standard statistical uncertainties, *u*, together with the Root Mean Square Deviation (RMSD) of the fit.

parameters	UiO-66		Al-UiO-66		DETA-UiO-66	
	value	*u*	value	*u*	value	*u*
q_m (mol·kg^{-1})	3.3895	0.1005	2.1727	0.1563	6.1585	13.1755
10^5 K (MPa^{-1})	5237740	655290	4609230	1361710	116100	288230
RMSD (mol·kg^{-1})	0.044		0.062		0.066	

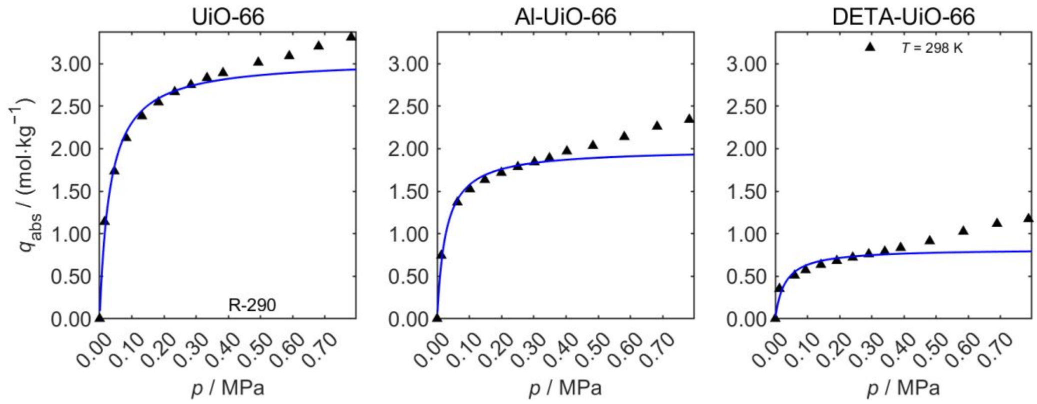

Figure S22: Absolute sorption capacity q_{abs} of the pure refrigerant R-290 on UiO-66, Al-UiO-66, and DETA-UiO-66 at different pressures p. ▲, T = 298 K. Pressure range: 0 ~ 0.79 MPa. The solid curves are the fits of the Langmuir model to the experimental data.

Table S20. Langmuir parameters of R-290 on three materials (UiO-66, Al-UiO-66, and DETA-UiO-66) and their standard statistical uncertainties, *u*, together with the Root Mean Square Deviation (RMSD) of the fit.

parameters	UiO-66		Al-UiO-66		DETA-UiO-66	
	value	*u*	value	*u*	value	*u*
q_m (mol·kg^{-1})	3.0514	0.1267	1.9959	0.0936	0.8220	0.0920
10^5 K (MPa^{-1})	3087750	644990	3729850	1054070	3372150	2114230
RMSD (mol·kg^{-1})	0.067		0.053		0.049	

3.3 Adsorption data for MIL-100(Fe) samples

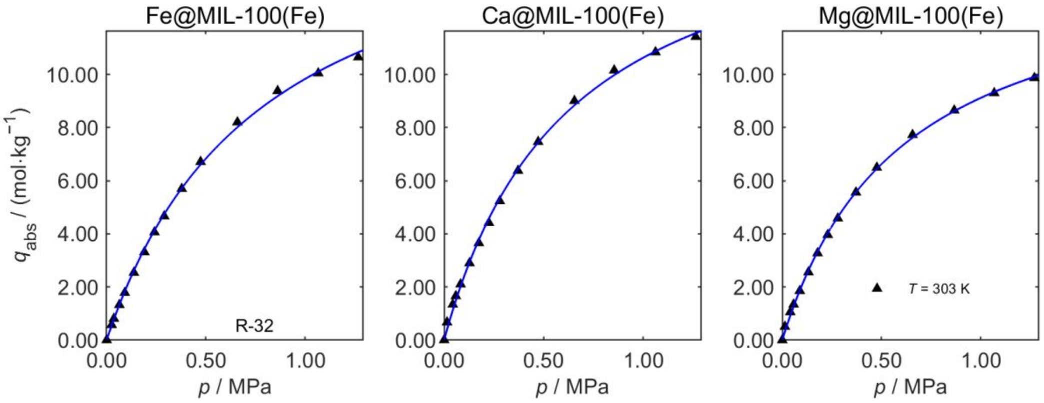

Figure S23: Absolute sorption capacity q_{abs} of the pure refrigerant R-32 on Fe@MIL-100(Fe), Ca@MIL-100(Fe), and Mg@MIL-100(Fe) at different pressures p. ▲, T = 303 K. Pressure range: 0 ~ 1.28 MPa. The solid curves are the fits of the Langmuir model to the experimental data.

Table S21. Langmuir parameters of R-32 on three materials (Fe@MIL-100(Fe), Ca@MIL-100(Fe), and Mg@MIL-100(Fe)) and their standard statistical uncertainties, u, together with the Root Mean Square Deviation (RMSD) of the fit.

parameters	Fe@MIL-100(Fe)		Ca@MIL-100(Fe)		Mg@MIL-100(Fe)	
	value	u	value	u	value	u
q_m (mol·kg^{-1})	17.5221	0.7009	17.2550	0.7554	14.6227	0.4004
10^5 K (MPa^{-1})	127930	9590	160480	14260	165720	9330
RMSD (mol·kg^{-1})	0.100		0.137		0.075	

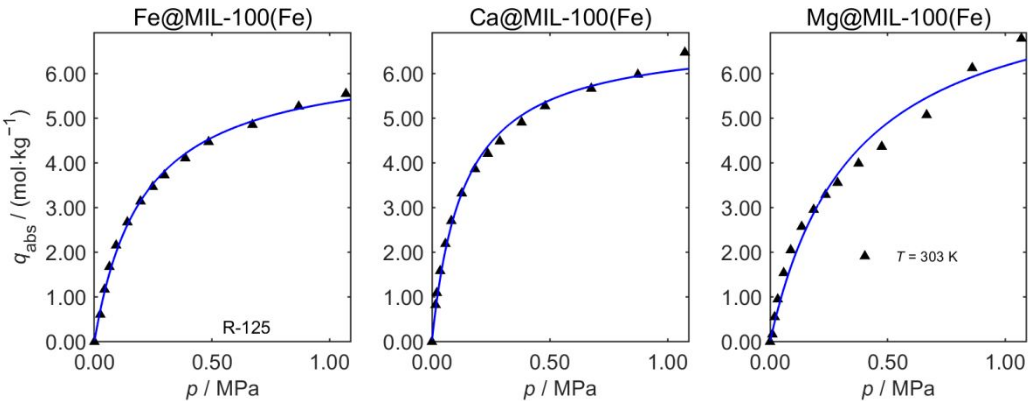

Figure S24: Absolute sorption capacity q_{abs} of the pure refrigerant R-125 on Fe@MIL-100(Fe), Ca@MIL-100(Fe), and Mg@MIL-100(Fe) at different pressures p. ▲, T = 303 K. Pressure range: 0 ~ 1.08 MPa. The solid curves are the fits of the Langmuir model to the experimental data.

Table S22. Langmuir parameters of R-125 on three materials (Fe@MIL-100(Fe), Ca@MIL-100(Fe), and Mg@MIL-100(Fe)) and their standard statistical uncertainties, u, together with the Root Mean Square Deviation (RMSD) of the fit.

parameters	Fe@MIL-100(Fe)		Ca@MIL-100(Fe)		Mg@MIL-100(Fe)	
	value	u	value	u	value	u
q_m (mol·kg^{-1})	6.4477	0.3071	6.8700	0.2951	8.3654	1.0908
10^5 K (MPa^{-1})	487040	65260	739350	103690	282730	85020
RMSD (mol·kg^{-1})	0.119		0.149		0.274	

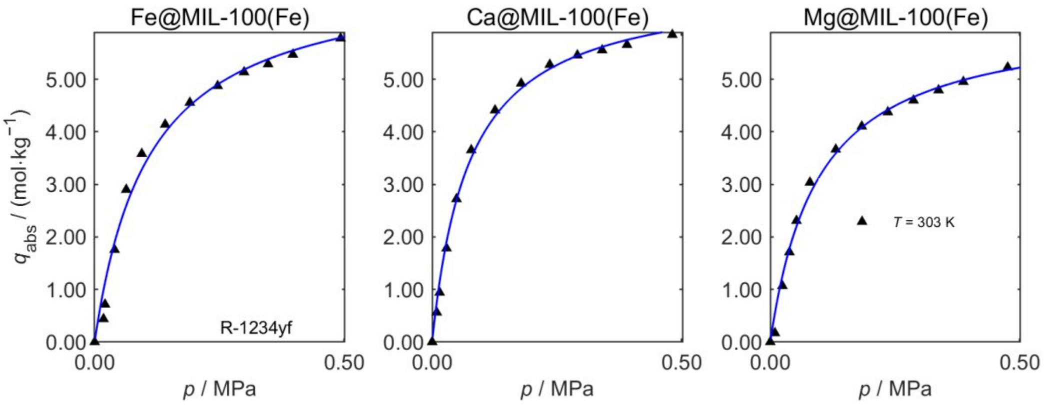

Figure S25: Absolute sorption capacity q_{abs} of the pure refrigerant R-1234yf on Fe@MIL-100(Fe), Ca@MIL-100(Fe), and Mg@MIL-100(Fe) at different pressures p. ▲, T = 303 K. Pressure range: 0 ~ 0.50 MPa. The solid curves are the fits of the Langmuir model to the experimental data.

Table S23. Langmuir parameters of R-1234yf on three materials (Fe@MIL-100(Fe), Ca@MIL-100(Fe), and Mg@MIL-100(Fe)) and their standard statistical uncertainties, u, together with the Root Mean Square Deviation (RMSD) of the fit.

parameters	Fe@MIL-100(Fe)		Ca@MIL-100(Fe)		Mg@MIL-100(Fe)	
	value	u	value	u	value	u
q_m (mol·kg^{-1})	7.0704	0.6529	6.8502	0.2440	6.2249	0.3638
10^5 K (MPa^{-1})	915110	249150	1332890	163560	1037290	185530
RMSD (mol·kg^{-1})	0.228		0.109		0.137	

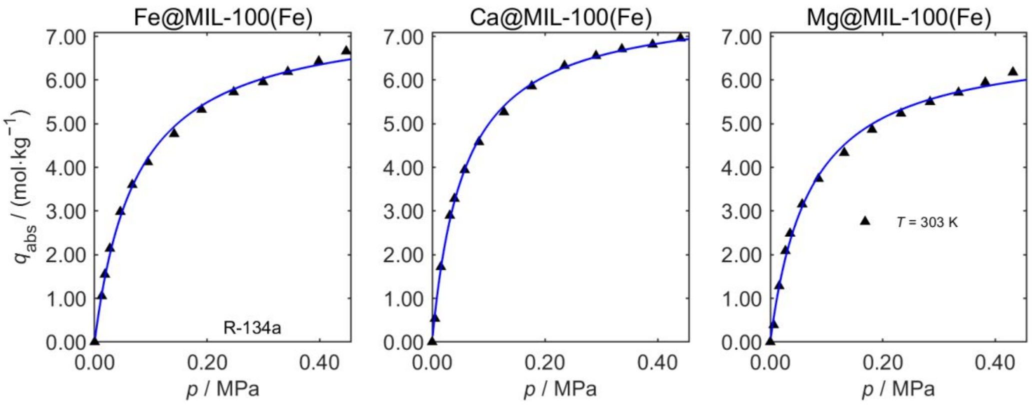

Figure S26: Absolute sorption capacity q_{abs} of the pure refrigerant R-134a on Fe@MIL-100(Fe), Ca@MIL-100(Fe), and Mg@MIL-100(Fe) at different pressures p. ▲, T = 303 K. Pressure range: 0 ~ 0.45 MPa. The solid curves are the fits of the Langmuir model to the

experimental data.

Table S24. Langmuir parameters of R-134a on three materials (Fe@MIL-100(Fe), Ca@MIL-100(Fe), and Mg@MIL-100(Fe)) and their standard statistical uncertainties, u, together with the Root Mean Square Deviation (RMSD) of the fit.

parameters	Fe@MIL-100(Fe)		Ca@MIL-100(Fe)		Mg@MIL-100(Fe)	
	value	u	value	u	value	u
q_m (mol·kg^{-1})	7.5420	0.2095	7.8011	0.1188	6.9367	0.2491
10^5 K (MPa^{-1})	1337990	123710	1772720	98420	1419840	172170
RMSD (mol·kg^{-1})	0.099		0.065		0.118	

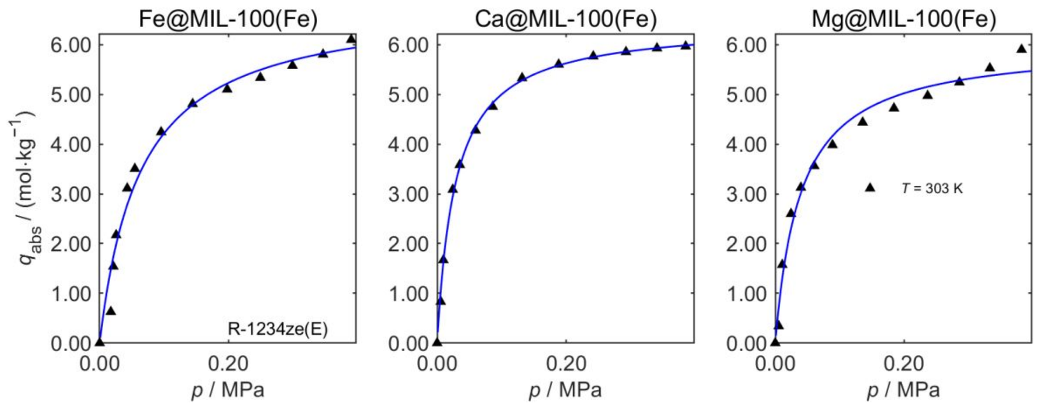

Figure S27: Absolute sorption capacity q_{abs} of the pure refrigerant R-1234ze(E) on Fe@MIL-100(Fe), Ca@MIL-100(Fe), and Mg@MIL-100(Fe) at different pressures p. ▲, T = 303 K. Pressure range: 0 ~ 0.40 MPa. The solid curves are the fits of the Langmuir model to the experimental data.

Table S25. Langmuir parameters of R-1234ze(E) on three materials (Fe@MIL-100(Fe), Ca@MIL-100(Fe), and Mg@MIL-100(Fe)) and their standard statistical uncertainties, u, together with the Root Mean Square Deviation (RMSD) of the fit.

parameters	Fe@MIL-100(Fe)		Ca@MIL-100(Fe)		Mg@MIL-100(Fe)	
	value	u	value	u	value	u
q_m (mol·kg^{-1})	6.8905	0.5955	6.4289	0.1158	6.0132	0.3936
10^5 K (MPa^{-1})	1576290	447790	3500950	282190	2530520	664300
RMSD (mol·kg^{-1})	0.280		0.078		0.227	

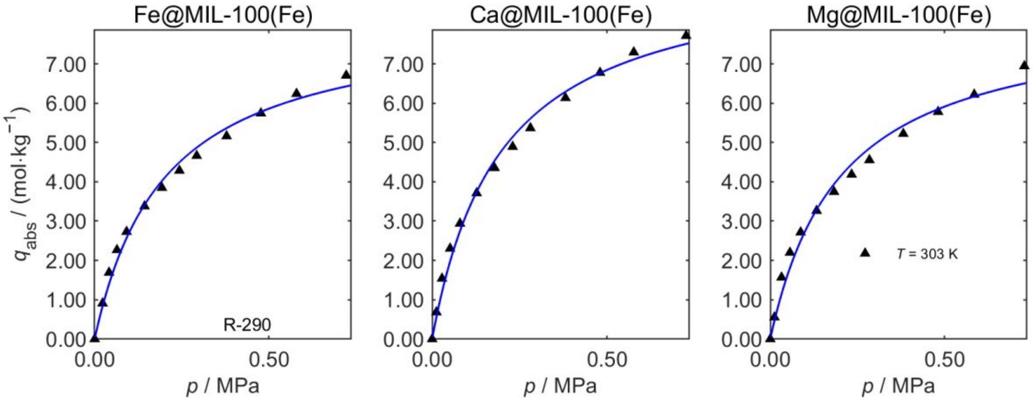

Figure S28: Absolute sorption capacity q_{abs} of the pure refrigerant R-290 on Fe@MIL-100(Fe), Ca@MIL-100(Fe), and Mg@MIL-100(Fe) at different pressures p. ▲, T = 303 K. Pressure range: 0 ~ 0.73 MPa. The solid curves are the fits of the Langmuir model to the experimental data.

Table S26. Langmuir parameters of R-290 on three materials (Fe@MIL-100(Fe), Ca@MIL-100(Fe), and Mg@MIL-100(Fe)) and their standard statistical uncertainties, u, together with the Root Mean Square Deviation (RMSD) of the fit.

parameters	Fe@MIL-100(Fe)		Ca@MIL-100(Fe)		Mg@MIL-100(Fe)	
	value	u	value	u	value	u
q_m (mol·kg^{-1})	8.2207	0.6457	9.5448	0.6926	8.3115	0.8735
10^5 K (MPa^{-1})	496510	99800	506830	95900	492890	133330
RMSD (mol·kg^{-1})	0.181		0.196		0.243	

3.4 Adsorption data for activated carbon samples

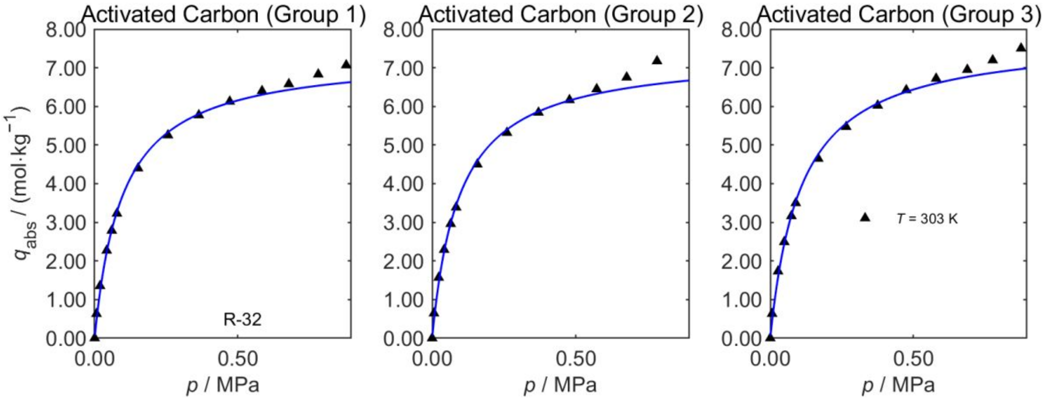

Figure S29: Absolute sorption capacity q_{abs} of the pure refrigerant R-32 on three groups of activated carbon at different pressures p. ▲, T = 303 K. Pressure range: 0 ~ 0.89 MPa. The solid curves are the fits of the Langmuir model to the experimental data.

Table S27. Langmuir parameters of R-32 on three groups of activated carbon and their standard statistical uncertainties, u, together with the Root Mean Square Deviation (RMSD) of the fit.

parameters	activated carbon (Group 1)		activated carbon (Group 2)		activated carbon (Group 3)	
	value	u	value	u	value	u
q_m (mol·kg^{-1})	7.3476	0.2176	7.3859	0.2226	7.8268	0.2647
10^5 K (MPa^{-1})	1033170	96680	1043990	99780	927250	95350
RMSD (mol·kg^{-1})	0.083		0.086		0.095	

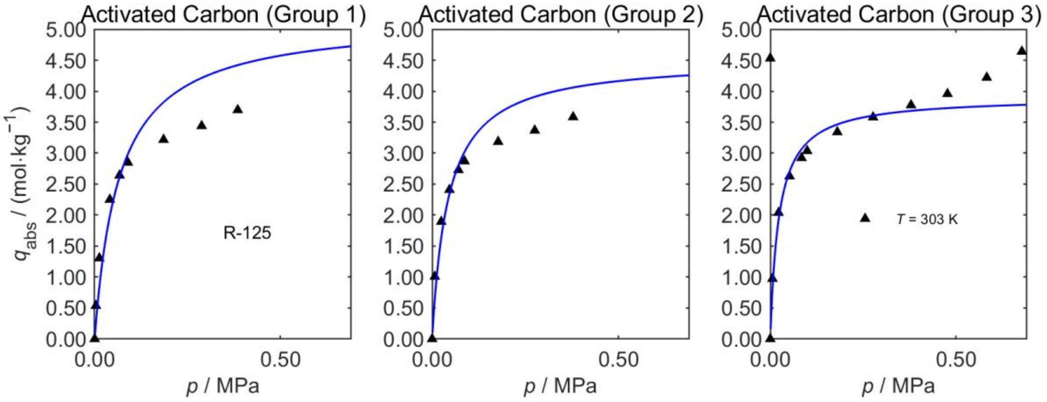

Figure S30: Absolute sorption capacity q_{abs} of the pure refrigerant R-125 on three groups of activated carbon at different pressures p. ▲, T = 303 K. Pressure range: 0 ~ 0.68 MPa. The solid curves are the fits of the Langmuir model to the experimental data.

Table S28. Langmuir parameters of R-125 on three groups of activated carbon and their standard statistical uncertainties, u, together with the Root Mean Square Deviation (RMSD) of the fit.

parameters	activated carbon (Group 1)		activated carbon (Group 2)		activated carbon (Group 3)	
	value	u	value	u	value	u
q_m (mol·kg^{-1})	5.1753	1.7968	4.5242	1.0553	3.9081	0.2033
10^5 K (MPa^{-1})	1522670	1829950	2309790	2071020	4319010	1156590
RMSD (mol·kg^{-1})	0.685		0.502		0.123	

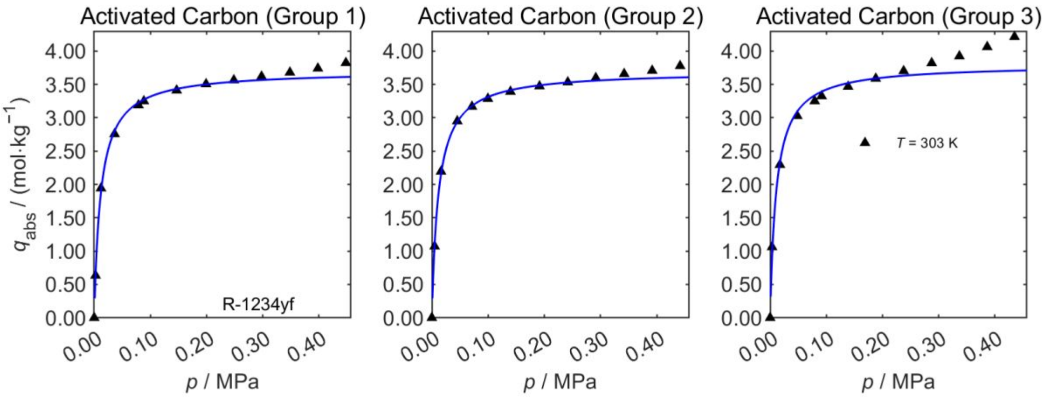

Figure S31: Absolute sorption capacity q_{abs} of the pure refrigerant R-1234yf on three groups of activated carbon at different pressures p. ▲, T = 303 K. Pressure range: 0 ~ 0.45 MPa. The

solid curves are the fits of the Langmuir model to the experimental data.

Table S29. Langmuir parameters of R-1234yf on three groups of activated carbon and their standard statistical uncertainties, *u*, together with the Root Mean Square Deviation (RMSD) of the fit.

parameters	activated carbon (Group 1)		activated carbon (Group 2)		activated carbon (Group 3)	
	value	*u*	value	*u*	value	*u*
q_m (mol·kg^{-1})	3.7059	0.0467	3.6998	0.0330	3.7986	0.1201
10^5 *K* (MPa^{-1})	8535860	677220	8530830	466160	9083410	1900860
RMSD (mol·kg^{-1})	0.032		0.023		0.084	

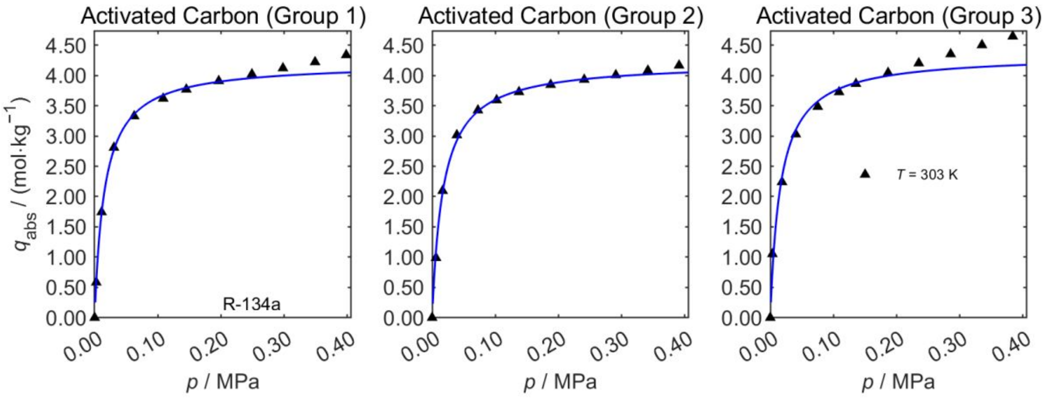

Figure S32: Absolute sorption capacity q_{abs} of the pure refrigerant R-134a on three groups of activated carbon at different pressures *p*. ▲, *T* = 303 K. Pressure range: 0 ~ 0.40 MPa. The solid curves are the fits of the Langmuir model to the experimental data.

Table S30. Langmuir parameters of R-134a on three groups of activated carbon and their standard statistical uncertainties, *u*, together with the Root Mean Square Deviation (RMSD) of the fit.

parameters	activated carbon (Group 1)		activated carbon (Group 2)		activated carbon (Group 3)	
	value	*u*	value	*u*	value	*u*
q_m (mol·kg^{-1})	4.2079	0.0593	4.2220	0.0929	4.3416	0.2287
10^5 *K* (MPa^{-1})	6338150	453750	5769520	607170	6170690	1694030
RMSD (mol·kg^{-1})	0.032		0.048		0.117	

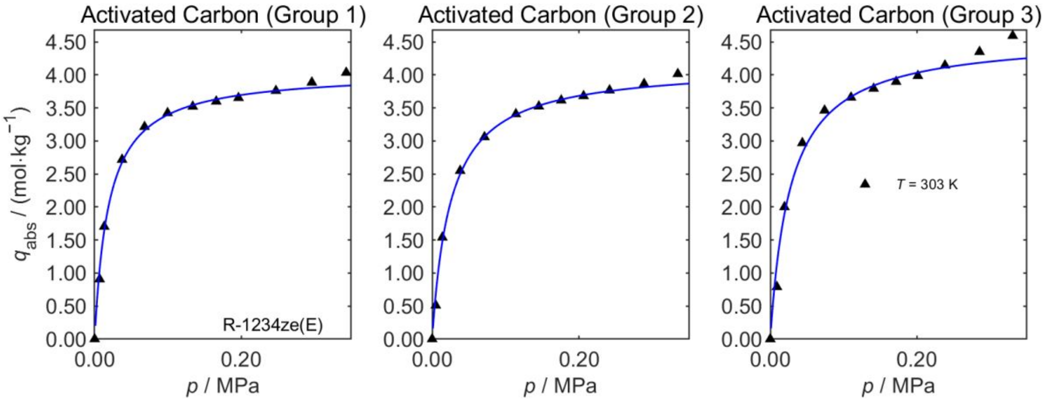

Figure S33: Absolute sorption capacity q_{abs} of the pure refrigerant R-1234ze(E) on three groups of activated carbon at different pressures *p*. ▲, T = 303 K. Pressure range: 0 ~ 0.35 MPa. The solid curves are the fits of the Langmuir model to the experimental data.

Table S31. Langmuir parameters of R-1234ze(E) on three groups of activated carbon and their standard statistical uncertainties, *u*, together with the Root Mean Square Deviation (RMSD) of the fit.

parameters	activated carbon (Group 1)		activated carbon (Group 2)		activated carbon (Group 3)	
	value	*u*	value	*u*	value	*u*
q_m (mol·kg^{-1})	4.0502	0.1261	4.1465	0.1503	4.5865	0.3377
10^5 K (MPa^{-1})	5200090	720640	3966730	606860	3628380	1050220
RMSD (mol·kg^{-1})	0.060		0.063		0.134	

4. Additional tables of sorption data

All the adsorption data in format of Adsorption Information File (AIF) [1], and in plain text format with uncertainty information are given in the zip files in the SI.

REFERENCES

[1] J. U. Keller and R. Staudt, *Gas adsorption equilibria: experimental methods and adsorptive isotherms*. Springer Science & Business Media, 2005.
